# Label-free optical metabolic imaging of adipose tissues for prediabetes diagnosis

**DOI:** 10.7150/thno.82697

**Published:** 2023-06-19

**Authors:** Liping Chen, Guihui Qin, Yuhong Liu, Moxin Li, Yue Li, Lun-Zhang Guo, Lidong Du, Weiming Zheng, Pei-Chun Wu, Yueh-Hsun Chuang, Xiaoyan Wang, Tzung-Dau Wang, Ja-An Annie Ho, Tzu-Ming Liu

**Affiliations:** 1 Institute of Translational Medicine, Faculty of Health Sciences, University of Macau, Macao SAR, China.; 2 MOE Frontiers Science Center for Precision Oncology, University of Macau, Macao SAR, China.; 3 School of Pharmaceutical Sciences, Guangzhou University of Chinese Medicine, Guangzhou, 510006, China.; 4 Institute of Biomedical Engineering, National Taiwan University, Taipei 10617, Taiwan.; 5 Translational Medicine R&D Center, Zhuhai UM Science and Technology Research Institute, Zhuhai, China.; 6 Department of Biochemical Science & Technology, National Taiwan University, Taipei 10617, Taiwan.; 7 Department of Anesthesiology, National Taiwan University Hospital and College of Medicine, Taipei 10002, Taiwan.; 8 Cardiovascular Center and Division of Cardiology, Department of Internal Medicine, National Taiwan University Hospital and College of Medicine, Taipei 10002, Taiwan.

**Keywords:** prediabetes, adipose tissue, redox ratio, NAD(P)H fluorescence lifetime, lipofuscin-like fluorescence

## Abstract

**Rationale:** Prediabetes can be reversed through lifestyle intervention, but its main pathologic hallmark, insulin resistance (IR), cannot be detected as conveniently as blood glucose testing. In consequence, the diagnosis of prediabetes is often delayed until patients have hyperglycemia. Therefore, developing a less invasive diagnostic method for rapid IR evaluation will contribute to the prognosis of prediabetes. Adipose tissue is an endocrine organ that plays a crucial role in the development and progression of prediabetes. Label-free visualizing the prediabetic microenvironment of adipose tissues provides a less invasive alternative for the characterization of IR and inflammatory pathology.

**Methods:** Here, we successfully identified the differentiable features of prediabetic adipose tissues by employing the metabolic imaging of three endogenous fluorophores NAD(P)H, FAD, and lipofuscin-like pigments.

**Results:** We discovered that 1040-nm excited lipofuscin-like autofluorescence could mark the location of macrophages. This unique feature helps separate the metabolic fluorescence signals of macrophages from those of adipocytes. In prediabetes fat tissues with IR, we found only adipocytes exhibited a low redox ratio of metabolic fluorescence and high free NAD(P)H fraction a_1_. This differential signature disappears for mice who quit the high-fat diet or high-fat-high-sucrose diet and recover from IR. When mice have diabetic hyperglycemia and inflamed fat tissues, both adipocytes and macrophages possess this kind of metabolic change. As confirmed with RNA-seq analysis and histopathology evidence, the change in adipocyte's metabolic fluorescence could be an indicator or risk factor of prediabetic IR.

**Conclusion:** Our study provides an innovative approach to diagnosing prediabetes, which sheds light on the strategy for diabetes prevention.

## Introduction

Type 2 diabetes mellitus (T2DM) accounts for up to 90% of all diabetes cases 1. By 2040, T2DM will affect around 642 million people worldwide, with an estimated health expenditure of 802 billion dollars 2. Most diagnostic methods for T2DM are based on glycaemic measures (fasting or 2-h postprandial glucose, or HbA_1C_) 3. Once the blood glucose test is abnormal, the patients have entered the diabetes stage, which requires lifelong management 4. Identifying and reversing diabetes at its early stage is therefore crucial. Before glucose changes can be detectable, impaired insulin sensitivity has already been present for 10 years 5, and insulin resistance (IR) is an important hallmark for the diagnosis of prediabetes 6. So far, the diagnostic methods for insulin sensitivity include the euglycemic insulin clamp (the gold standard) 7, the hyperglycemic clamp 7, the insulin suppression test 8, and tracer detection 9. But these detection methods are either invasive or time-consuming, which is inconvenient for prediabetes screening and monitoring.

At the prediabetic stage, several metabolic imbalances are already established. Identifying these imbalances with adequate and precise biomarkers can facilitate early intervention. Adipose tissue is the critical organ for the onset of IR and inflammation 10, 11. Overnutrition or obesity can cause excessive hypertrophy of adipocytes with decreased mitochondrial content and impaired mitochondrial biogenesis 12. Mitochondrial dysfunction leads to IR through impairing adipogenesis, lipolysis, oxidative phosphorylation (OXPHOS), and increasing free-fatty acid 13. Another critical player for adipose tissue inflammation is the activated macrophages. In diabetic rodents and individuals, approximately 90% of macrophages selectively infiltrate the site surrounding necrotic adipocytes, where they form crown-like structures (CLSs), phagocytose dead adipocytes, and actively produce pro-inflammatory cytokines 14. These metabolic and histopathologic changes in adipose tissues provide an opportunity to detect prediabetes. Conventionally, histopathology assessment relies on invasive biopsy and cumbersome dye-staining processes. Some people use positron emission tomography-computed tomography (PET/CT) 15, or magnetic resonance imaging (MRI) 16 to monitor the physiopathologic change of adipose tissues. However, they possess several limitations, including radioactivity, time-consuming, expensive, and, what's more, they require some exogenous contrast agents which may perturb the physiological condition of the tissue. Therefore, developing a label-free and non-destructive imaging of adipose tissue metabolism in prediabetes is extremely important.

The metabolic imaging of autofluorescent coenzymes reduced nicotinamide adenine dinucleotide (phosphate) (NAD(P)H), and oxidized flavin adenine dinucleotide (FAD), allow label-free detection of cellular or tissue metabolism. One can non-destructively map their spatial distribution with two-photon fluorescence microscopy (TPFM) and/or fluorescence lifetime imaging microscopy (FLIM). The calculated redox ratio and lifetime indices from these images enable label-free detection of neoplastic development 17, stem cell differentiation 18, macrophage polarization 19, the morphological and functional difference among various types of adipose tissues 20, and cellular response to environmental stimulations 21-23. However, the adipose tissues are multi-cellular environments, mainly composed of adipocytes and macrophages. They may exhibit different metabolic features in response to IR and inflammation. So far, no label-free method exists to identify the boundaries between them. Metabolic fluorescence from different types of cells will mix 24, and the prediabetic features may be neglected.

Previous studies have shown that macrophages contain aging-accumulated lipofuscin 25. Since lipofuscin autofluorescence has been used in diagnosing chronic liver disease 26, brain damage 27, and obesity 28, we speculate that macrophages' lipofuscin may have its characteristic autofluorescence. Here, we found the 1040-nm pulse laser could excite two-photon red autofluorescence specific to macrophages. We used this newly discovered feature to separate macrophages' NAD(P)H/FAD fluorescence from adipocytes in TPFM and FLIM images*.* Multivariate analyses demonstrated that macrophages and adipocytes exhibit different metabolic dynamics in the early and late stages of diabetes. Logistic regression models based on these optical parameters yield a predictive accuracy of approximately 0.9 (area under the receiver operating characteristic (ROC) curve (AUC)) for distinguishing between healthy and prediabetic mice. The differential metabolic features can be developed as a label-free and low-invasiveness method to screen prediabetes.

## Results

### Lipofuscin-like fluorescence label-free marks the spatial location of macrophages

**Figure 1:**
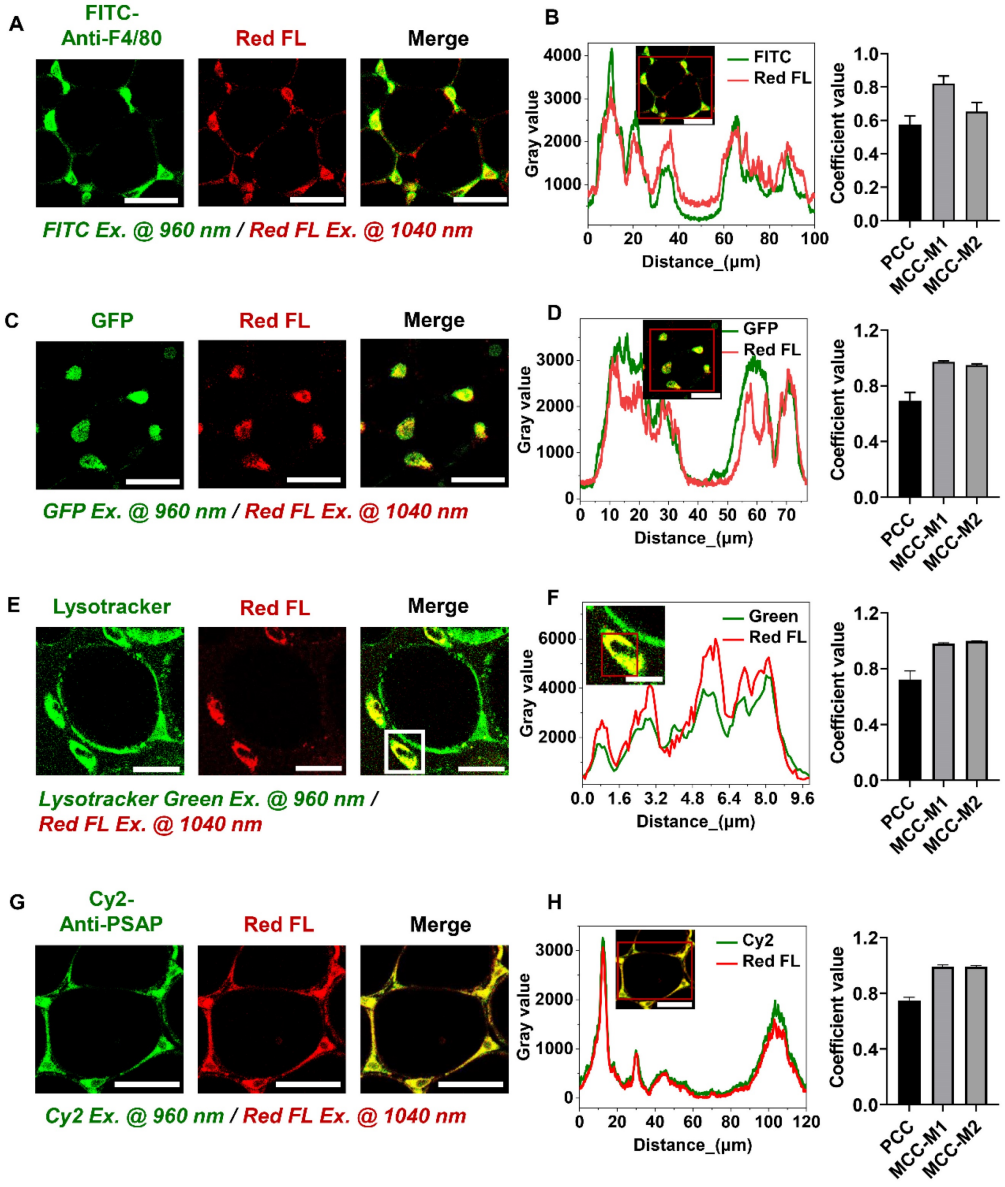
** Lipofuscin-like fluorescence can be used as a label-free marker for adipose tissue macrophages. (A)** Co-localization of lipofuscin-like red autofluorescence and macrophage marker F4/80 in the epididymal fat of C57 mice. FITC-anti-mouse F4/80 excited by 960 nm was presented by green color, and lipofuscin-like red autofluorescence (Red FL) excited by 1040 nm was presented by red color. Scale bar: 50 μm. **(B)** (left panel) Intensity profiles of FITC and lipofuscin-like red autofluorescence within the red rectangular region of the merged image (inset). (right panel) PCC = 0.57 ± 0.052, MCC-M1= 0.82 ± 0.045, and MCC-M2 = 0.65 ± 0.053. **(C)** Co-localization of lipofuscin-like red autofluorescence and GFP in the epididymal fat of C57BL/6J-c2J-LysM-eGFP mice. Scale bar: 50 μm.** (D)** (left panel) Intensity profiles of GFP and lipofuscin-like fluorescence within the red rectangular region of the merged image (inset). (right panel) PCC = 0.69 ± 0.058, MCC-M1 = 0.97 ± 0.008, MCC-M2 = 0.94 ± 0.01. **(E)** Co-localization of lipofuscin-like red autofluorescence and lysosome in the epididymal fat of C57 mice. Lysosome was identified with lysotracker green. Scale bar: 20 μm. **(F)** (left panel) Intensity profiles of lysotracker green fluorescence and lipofuscin-like red fluorescence within the red rectangular region of the merged image (inset). (right panel) PCC = 0.72 ± 0.061, MCC-M1 = 0.98 ± 0.005, MCC-M2 = 0.99 ± 0.0005. **(G)** Co-localization of lipofuscin-like red autofluorescence and lipofuscin marker PSAP in the epididymal fat of C57 mice. Scale bar: 50 μm. **(H)** (left panel) Intensity profiles of Cy2 and lipofuscin-like fluorescence within the red rectangular region of the merged image (inset). (right panel) PCC = 0.75 ± 0.024, MCC-M1 = 0.99 ± 0.01, MCC-M2 = 0.99 ± 0.007. Data in (B, D, F, H) are presented as Mean ± SD (n = 3). Red FL represents lipofuscin-like red autofluorescence.

During metabolic imaging of adipose tissues, we found that the regions where macrophages usually reside exhibit strong two-photon red autofluorescence excited at 1040 nm (**Red color in Figure 1A, C, E, G**). Good co-localization between FITC-conjugated anti-mouse F4/80 and red autofluorescence suggested that red autofluorescence may be specific to macrophages (**Figure 1A-B**). We further used C57BL/6J-c2J-LysM-eGFP transgenic mice 29 to confirm whether this characteristic red autofluorescence can specifically identify macrophages, in which the GFP expression is driven by the lysozyme promoter (**Figure S1A-B**). In the freshly isolated epididymal fat, we found GFP-positive cells emit 1040-nm excited red autofluorescence (**Figure 1C-D**). This feature also holds true for bone marrow-derived macrophages, which exhibited higher red autofluorescence intensity than 3T3-L1 cells (**Figure S1C-F**). These results indicate that 1040-nm excited two-photon red autofluorescence is a specific hallmark of macrophages. Intriguingly, the corresponding autofluorescence spectrum (**Figure S1G**) resembles the red emission band of aging-specific lipofuscins 30. Previous studies have demonstrated that macrophages contain lipofuscins 31, 32 that mainly accumulate in lysosomes 33. We thus speculated that the two-photon red autofluorescence might originate from lipofuscin pigments. So, we stained macrophages' lysosomes and confirmed their co-localization with red autofluorescence (**Figure 1E-F**). Immunostaining of lipofuscin in adipose tissue further verified that lipofuscin-positive cells emit 1040-nm excited red autofluorescence (**Figure 1G-H**). Altogether, the lipofuscin-like red autofluorescence enables non-destructively spatial localization of macrophages in adipose tissue, and this label-free technique can be used to separate the metabolic fluorescence of macrophages from those of adipocytes in the following investigations.

### Only adipocytes show a lower redox ratio and a larger proportion of free NAD(P)H fluorescence in prediabetic mice

**Figure 2:**
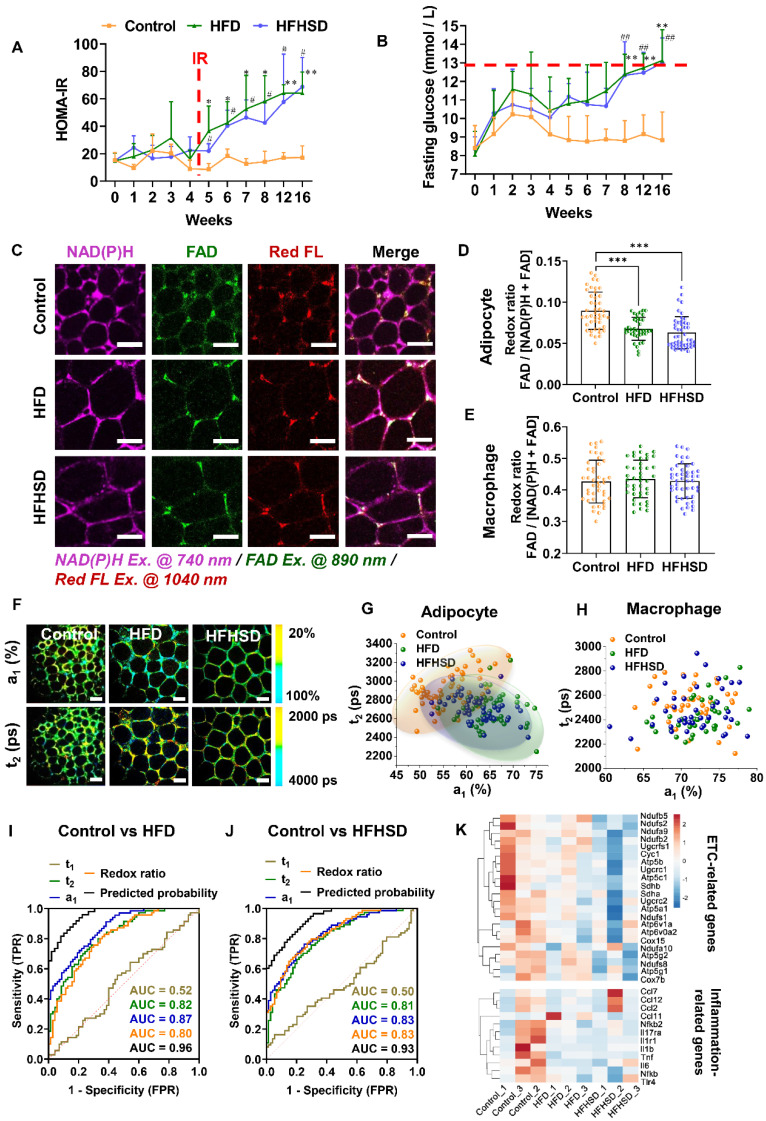
** Optical readouts of adipocytes and macrophages in response to the prediabetic microenvironment of epididymal adipose tissue. (A)** Changes of HOMA-IR index in mice fed HFD or HFHSD for 16 weeks. The red dotted line marks the development of IR in mice fed HFD or HFHSD for 5 weeks. **(B)** Fasting glucose levels in mice fed HFD or HFHSD for 16 weeks. The red dotted line marks the blood glucose higher than 13mmol/L in mice fed HFD or HFHSD for 16 weeks. **(C)** Representative images of NAD(P)H, FAD, and lipofuscin-like fluorescence intensity in epididymal fat from mice fed HFD or HFHSD for 1 month. Scale bar: 50 μm. **(D)** FAD/[NAD(P)H + FAD] intensity of adipocyte cytoplasm was quantified. **(E)** Quantification of redox ratio of adipose tissue macrophages. **(F)** Pseudo-color-coded FLIM a_1_ and t_2_ images of NAD(P)H in adipose tissues. Scale bar: 50 μm. **(G)** The a_1_-t_2_ scatter plot of adipocytes. Control: orange, HFD: olive, and HFHSD: royal. **(H)** The a_1_-t_2_ scatter plot of macrophages. **(I, J)** ROC curves and AUC values for optical readouts (redox ratio, a_1_, t_1_, t_2_, and t_2_-a_1_-redox ratio-integrated parameter) of adipocytes, showing their ability to distinguish between **(I)** control and HFD groups or between **(J)** control and HFHSD groups. **(K)** Expression profiles of OXPHOS-related and inflammation-related genes in adipose tissues (n = 3). Data in (A, B, D, E) are expressed as Mean ± SD (n = 8). ^*^P < 0.05, ^**^P < 0.01, and ^***^P < 0.001, the HFD group *vs.* the control group;^ #^*P* < 0.05, ^##^*P* < 0.01, and ^###^*P* < 0.001, the HFHSD group *vs.* the control group. Each group in (D, E, G, H) contains 25-30 data points from 8 biological replicas. Each data point is the average of 10 cells in one field of view.

Our animal models used a high-fat diet (HFD) or high-fat-high-sucrose diet (HFHSD) to induce diabetes conditions. Mice developed prediabetic IR after 1 month, whereas diabetic hyperglycemia (above 13 mmol/L) was not observed until the 4^th^ month (**Figure 2A-B, Figure S2A-J**). Taking advantage of the lipofuscin-laden feature of macrophages, we could label-free analyze the NAD(P)H and FAD metabolic fluorescence of macrophages and adipocytes separately in adipose tissues. In prediabetic mice, we found that only adipocytes showed a significant decrease in redox ratio in freshly isolated epididymal fats (**Figure 2C-E**). The NAD(P)H FLIM of adipose tissues exhibited a shortened NAD(P)H lifetime t_2_ and an increased a_1_ in adipocytes (**Figure 2F-G**) but not in macrophages (**Figure 2H**). A possible cause of increased NAD(P)H fraction (a_1_) and shorter protein-bound NAD(P)H lifetime (t_2_) is a higher concentration ratio of NADH over NADPH due to a malfunctioning electron transport chain (ETC) and an overactive polyol pathway in the prediabetic or diabetic microenvironment 34-38. The utilization of a_1_ and t_2_ indices yielded an original classification accuracy of 92.3% (control *vs.* HFD) or 81.8% (control *vs.* HFHSD) and a cross-validation classification accuracy of 91.3% (control *vs.* HFD) or 80.9% (control *vs.* HFHSD). Compared with the redox ratio, lifetime indicators could better differentiate the metabolic state of adipocytes between healthy and prediabetic mice. The integrated indices of redox ratio, a_1_, and t_2_ could differentiate between healthy and prediabetic mice with a high sensitivity (control *vs.* HFD: 97%; control *vs.* HFHSD: 89%), 100% specificity, and high AUC accuracy (control *vs.* HFD: 0.96; control *vs.* HFHSD: 0.93) (**Figure 2I-J, Table S1**). Moreover, the AUC of disease prediction probability obtained by multi-parameter analysis is higher than that of single-parameter AUC, indicating that the diagnostic ability of multi-dimensional optical indices is more advantageous than that of a single parameter.

Here in prediabetes conditions, decreased redox ratio and increased a_1_ may be the result of reduced OXPHOS or elevated glycolysis 39-43. To clarify the underlying mechanism, we specifically examined genes related to metabolic pathways through RNA-Seq of adipose tissues. Several genes involved in oxidative metabolism, including PGC-1α, and ETC genes, were downregulated in prediabetic mice, but glycolysis-related and pro-inflammatory-related genes were not significantly changed (**Figure 2K, Figure S3A**). The Seahorse assay revealed a decrease in oxygen consumption rate (OCR) and no significant difference in extracellular acidification rate (ECAR) in prediabetic adipose tissue compared to the controls (see **Figure S3B**-**C**). These results are consistent with the RNA-seq data, suggesting impaired oxidative metabolism and mitochondrial dysfunction in prediabetic adipose tissue. Based on these findings, we can reasonably speculate that decreased redox ratio, increased a_1,_ and shortened t_2_ may be attributed to mitochondrial dysfunction and reduced OXPHOS. Moreover, in line with the optical metrics and RNA-seq outcomes, the results of Hematoxylin and Eosin (H&E) staining and immunohistochemical (IHC) staining further validated the absence of variation in CD80, iNOS (typical markers of M1 macrophage) and CD206 expression (a typical marker of M2 macrophage) between normal and prediabetic adipose tissues (**Figure S3D**). These results support our findings in optical metrics. Therefore, measuring the autofluorescence intensities of lipofuscin, NAD(P)H, and FAD, as well as the fluorescence lifetime of NAD(P)H, could sensitively and specifically detect the IR of prediabetes.

### Both adipocytes and macrophages exhibit abnormal metabolic fluorescence in diabetic mice

**Figure 3:**
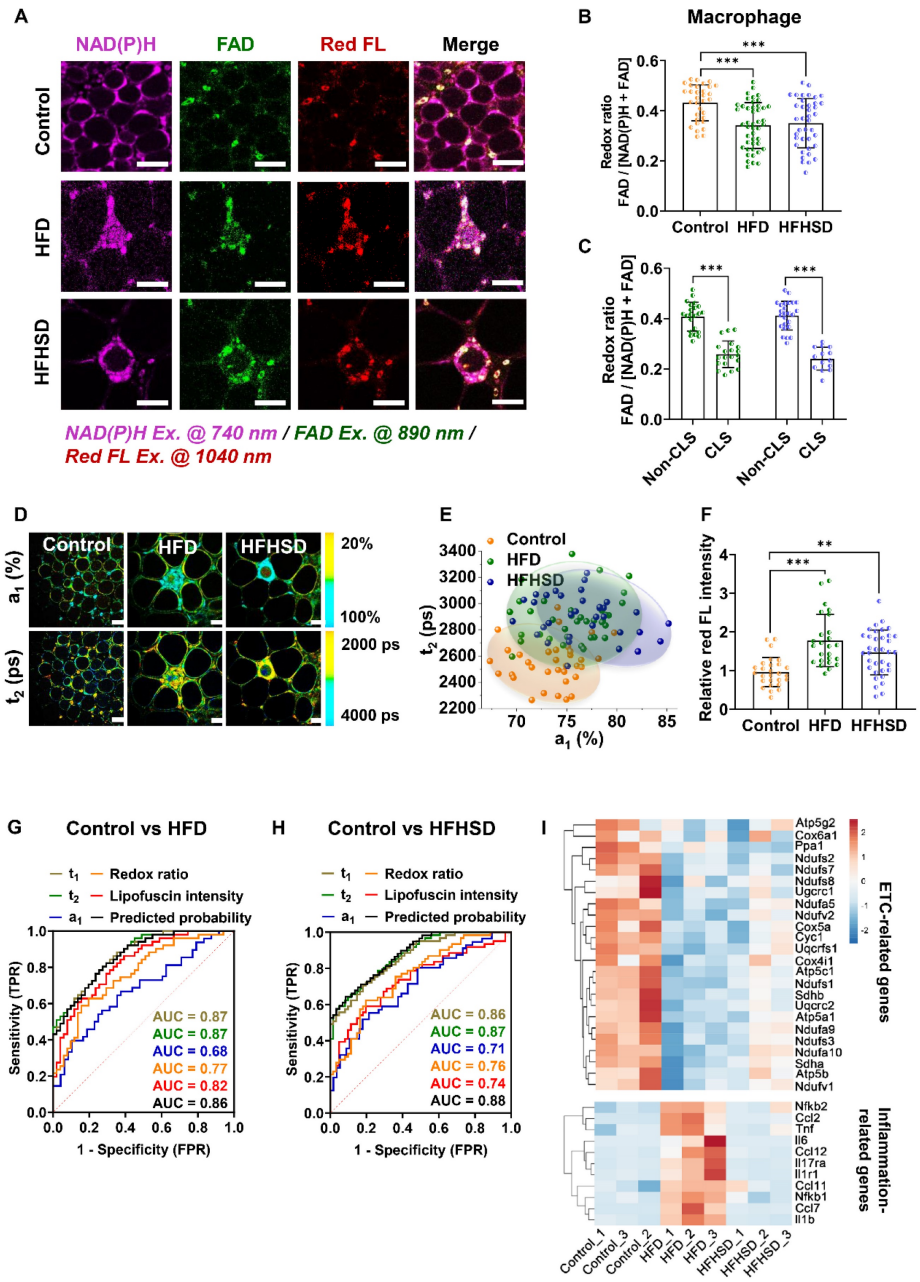
** TPFM and FLIM metabolic imaging of epididymal adipose tissues of diabetic mice. (A)** Representative images of NAD(P)H, FAD, and lipofuscin-like fluorescence intensity in epididymal adipose tissues from mice fed HFD or HFHSD for 4 months. Scale bar: 50 μm. **(B)** Quantification of macrophages' redox ratio.** (C)** The redox ratio of macrophages in CLSs and non-CLSs (Left: HFD group; right: HFHSD group) was quantified. **(D)** Pseudo-color-coded FLIM a_1_ and t_2_ images of NAD(P)H in adipose tissues. Scale bar: 50 μm. **(E)** The a_1_-t_2_ scatter plot of macrophages. **(F)** Quantification of the relative fluorescence intensity of lipofuscin-like pigments in macrophages. **(G, H)** ROC curves and AUC values for optical readouts (redox ratio, a_1_, t_1_, t_2_, lipofuscin intensity, and t_2_- t_1_-a_1_-redox ratio-lipofuscin-integrated parameter) of macrophages, showing their ability to distinguish between** (G)** control and HFD groups or between **(H)** control and HFHSD groups. **(I)** Heatmap of OXPHOS-related and inflammation-related genes expression in adipose tissues of normal and diabetic mice. Data in (B, C, F) are presented as Mean ± SD (n = 8). ^**^P < 0.01 and ^***^P < 0.001 relative to the control group. Each group in (B, C, E, and F) contains 25-30 data points from 8 biological replicas. Each data point is the average of 10 cells in one field of view.

In contrast to prediabetic mice, decreased redox ratios were found in both adipocytes and macrophages of diabetic mice (**Figure 3A-B, Figure S4**). Interestingly, compared with the control mice, more CLSs (red arrows) appeared in the adipose tissue of diabetic mice, especially the HFD-fed mice (**Figure S5A**). Macrophages that aggregate into the CLS stained positive for CD80 and iNOS (the M1 macrophage markers) (**Figure S5B-F**). Their redox ratios were significantly lower than that of non-CLS macrophages (**Figure 3C**), indicating that CLS macrophages are the main contributor to the redox ratio reduction. In addition, both adipocytes and macrophages of diabetic mice exhibited increased lifetime proportion a_1_ of free NAD(P)H (**Figure 3D, Figure S4**). The a_1_-t_2_ scatter plot distinctly separated macrophages in the diabetic mice from those in the control group, with a high original (86.1% for HFD vs control; 88.7% for HFHSD vs control) and cross-validation accuracy (86.1% for HFD vs control, 84.5% for HFHSD vs control) (**Figure 3E**). Moreover, higher lipofuscin intensity was observed in macrophages from diabetic mice, especially macrophages in CLSs (**Figure 3F**), which may imply a pro-inflammatory status 44-46. To visually compare the accuracies of optical indices in classifying the metabolic profile of different macrophage phenotypes, we plotted the ROC curves of the redox ratio, a_1_, t_1_, t_2_, and the relative lipofuscin-like fluorescence intensity. The AUC analysis yielded good separation of datasets between healthy and diabetic mice with accuracies of 0.86 and 0.88 for HFD vs control and HFHSD vs control, respectively (**Figure 3G-H, Table S2-3**). As an *in vitro* validation, we isolated mice's bone marrow-derived macrophages (BMDMs) and stimulated them into M1 and M2 phenotypes. Consistent with the results in adipose tissues, BMDM M1 macrophages had a lower redox ratio and higher a_1_ and t_2_ compared to BMDM M0 and M2 macrophages (**Figure S6**). The discrepancies in the absolute values of macrophage's redox ratio *in vivo* and* in vitro* may be due to the more complex microenvironment *in vivo*. For example. adipocytes could transfer mitochondria to macrophages in adipose tissues to maintain metabolic homeostasis 47. Moreover, RNA-Seq analysis on the adipose tissues successfully identified the down-regulation of ETC-related genes and up-regulation of several inflammation-related genes including TNF-α, interleukins (ILs), CC chemokines, and NF-κB in diabetic mice (**Figure 3I**), while changes in glycolysis-related genes' expression were not obvious (**Figure S7A**). In addition, the Seahorse assay demonstrated that compared with the normal group, the OCR of adipose tissue was lower in diabetic mice, while the ECAR remained unchanged. This is consistent with the RNA-Seq result (**Figure S7B and C**). So, based on these results, we thought reduced OXPHOS could be a major cause of abnormal metabolic fluorescence in the diabetic microenvironment. This further proved the reliability of our newly-developed approach for label-free characterization of macrophage phenotypes and diagnosis of diabetic adipose tissues *in situ*.

### Integrated analysis of metabolic imaging readouts: a promising approach for differential diagnosis of diabetes stages

**Figure 4:**
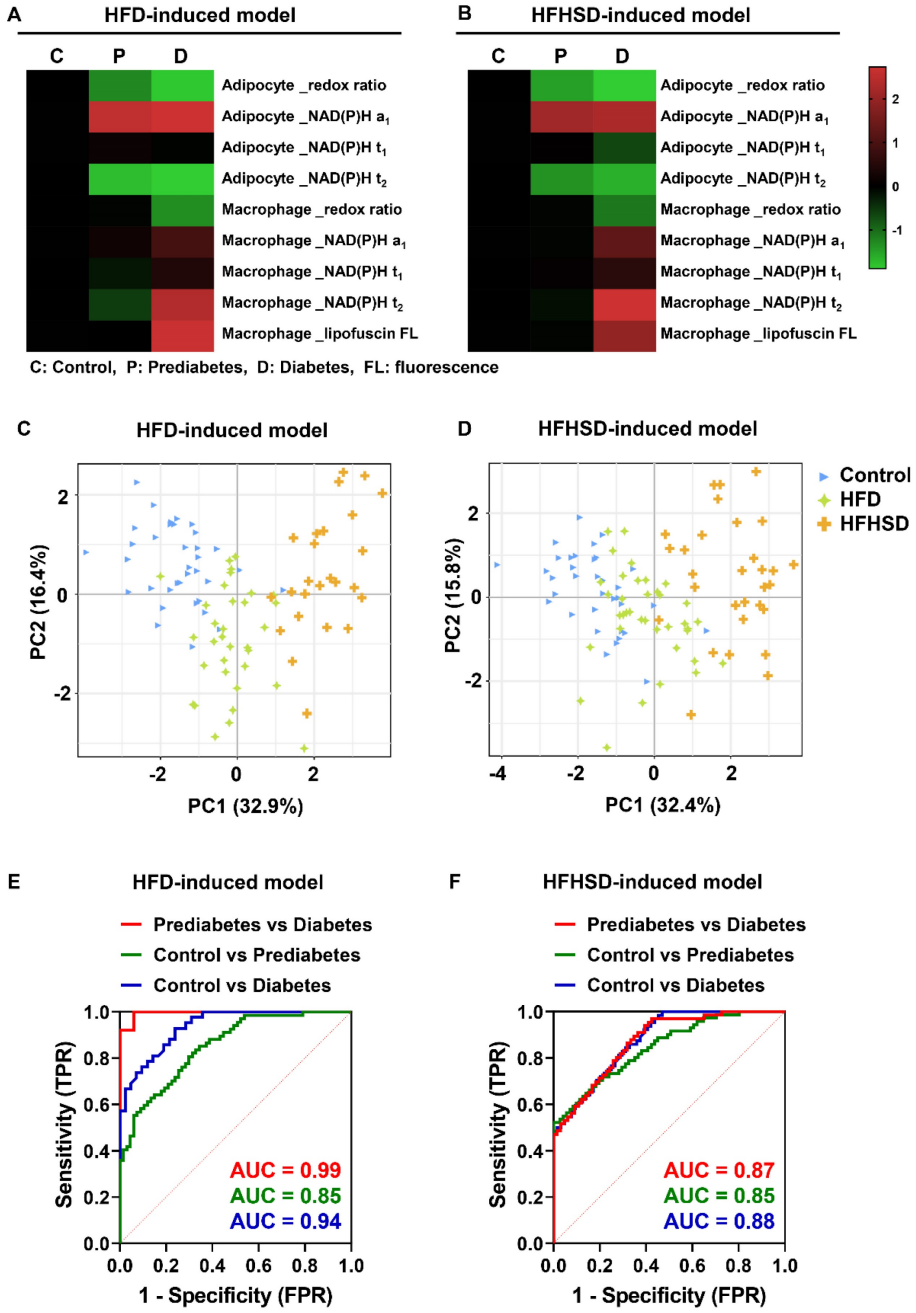
** Integrated analysis of metabolic imaging readouts enables the differential diagnosis of diabetes stages. (A, B)** Z-score heatmaps showing changes in optical readouts of adipocytes and macrophages in healthy control, prediabetes, and diabetes conditions. **(C, D)** PCA scores plots (PC1 versus PC2) for the differentiation of integrated metabolic imaging readouts from control, prediabetic (HFD and HFHSD), and diabetic (HFD and HFHSD) mice. For each group, 25-30 data points were obtained from 8 biological replicas. **(E, F)** ROC curves based on the optical metrics of adipose tissues for differential diagnosis of prediabetes versus diabetes, control versus prediabetes, and control versus diabetes.

We then compared the metabolic imaging readouts of control, prediabetic, and diabetic adipose tissues to explore their potential for differential diagnosis. The Z-score heatmaps of nine optical variables showed clear differences in metabolic fluorescence between normal, prediabetic, and diabetic adipose tissues (**Figure 4A**-**B**, **Table S4**). Principal component analysis (PCA) score plots indicated that the metabolic autofluorescence of these three groups was well-clustered and separated in both the HFD and HFHSD models (**Figure 4C**-**D**), with high accuracy (AUC) greater than 0.85 (**Figure 4E**-**F**, **Table S5**). Thus, a multi-optical readout analysis of adipocytes and macrophages in adipose tissue can enable differential diagnosis among normal, prediabetic, and late diabetes stages.

### Remission of IR eliminates the differential features of metabolic fluorescence

**Figure 5:**
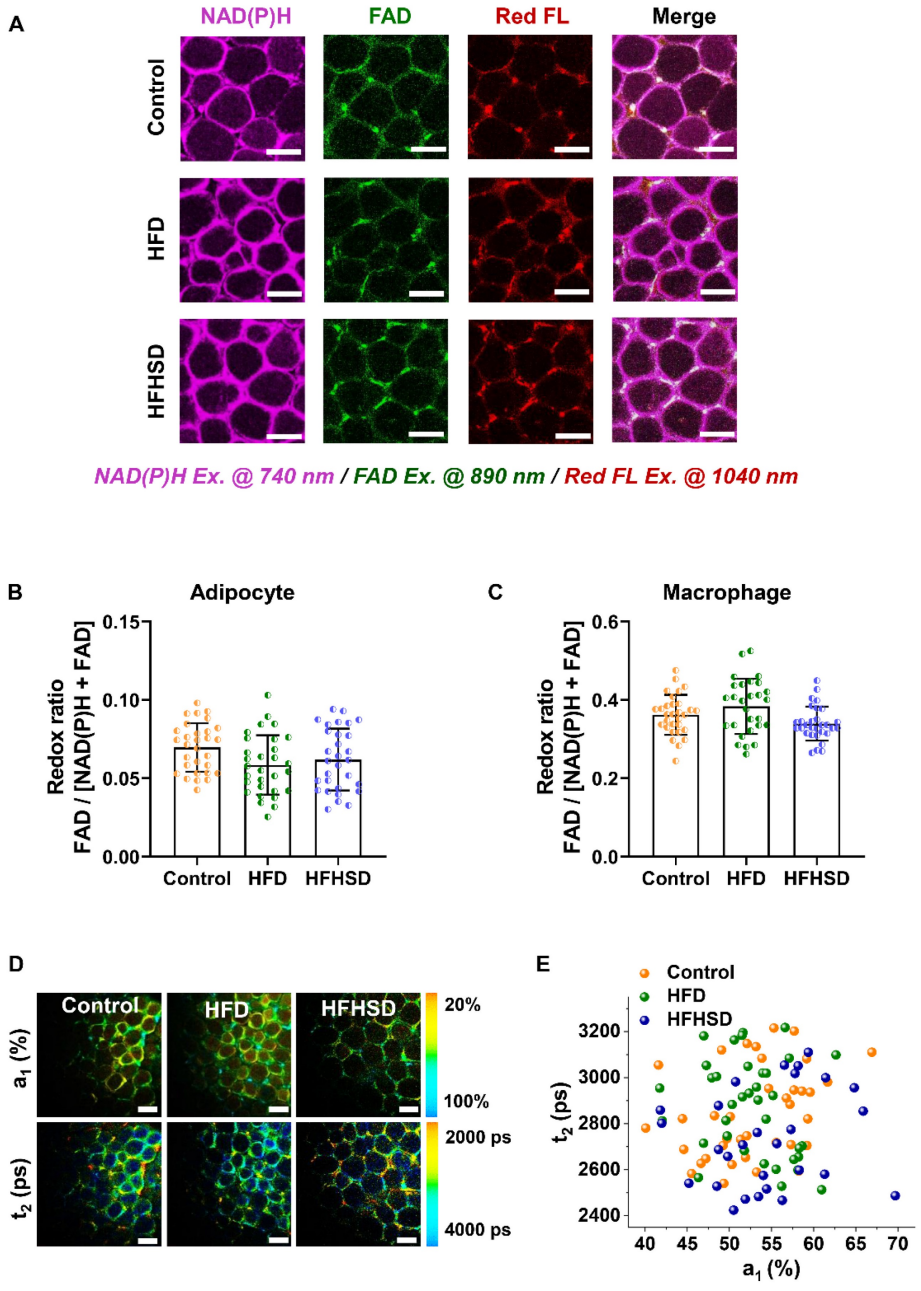
** Remission of IR in mice restoring the redox ratio and NAD(P)H lifetime of adipose tissue to normal levels. (A)** Representative images of NAD(P)H, FAD, and lipofuscin fluorescence intensity. Scale bar: 50 μm. Relative FAD/[NAD(P)H + FAD] intensities in adipocyte cytoplasm **(B)** and macrophages **(C)** in adipose tissues were quantified. **(D)** False color-coded FLIM a_1_ and t_2_ images of NAD(P)H in adipose tissues. Scale bar: 50 μm. **(E)** The a_1_-t_2_ scatter plot showed that no difference in NAD(P)H lifetime of adipocytes was observed among the three groups. Data are shown as Mean ± SD (n = 8). Each group in (B, C, and E) contains 25-30 data points from 8 biological replicas. Each data point is the average of 10 cells in one field of view.

Overwhelming evidence has shown that lifestyle interventions, especially improvements in physical activity and diet, can prevent or delay the progression of diabetes 48-50. To prove that the metabolic shift of adipocytes results from IR, we performed rescue experiments by converting HFD or HFHSD into a normal chow diet after IR (**Figure S8A**). Several notable changes were observed in mice after changing the diet. Blood glucose levels of prediabetic mice had returned to normal (**Figure S8B-C**). Additionally, histopathological examination manifested that dietary improvement in prediabetic mice normalized adipocyte size and reversed inflammatory conditions, such as the reduction of CLS numbers (**Figure S8D-E**). More importantly, the metabolic changes of redox ratio and NAD(P)H lifetime that we found in prediabetes disappeared (**Figure S9, Figure 5A-E**). These results not only suggest that dietary change is an effective way to prevent the deterioration of prediabetes and the development of diabetes, but also demonstrate that these optical parameters can be used as reliable indices to detect IR and inflammation in prediabetic and diabetic microenvironments.

## Discussion

Adipose tissue is an endocrine organ that plays a pivotal role in the development of IR. Dysfunction of adipose tissue releases more adipokines, pro-inflammatory factors, and free fatty acids to disrupt the balance of glucose and lipid metabolism 51, 52 and impair insulin signaling transduction 53, 54. At present, there are limited ways to monitor adipose tissue metabolism during the presymptomatic stage of T2DM, especially the label-free methods. Here, we demonstrated that triple metabolic imaging of NAD(P)H, FAD, and lipofuscin-like autofluorescence could label-free identify the metabolic heterogeneity of adipose tissues in the prediabetic and diabetic microenvironment. The key advance, newly discovered lipofuscin-like fluorescence, could separate macrophages from adipocytes. This identity separation technique allows us to find the redox ratio and NAD(P)H lifetime has differentiable features in the adipocytes of prediabetic IR as well as distinguishable features in the M1-activated macrophages (**Figures 2, 3, S5, and S6**).

Lipofuscin pigments are a kind of chemically and morphologically polymorphic waste consisting of cross-linked lipids, protein residues, and other undigested cell substances that accumulate primarily in lysosomes 33. They commonly appear in senescent cells 55 and have a broad excitation range from 320 to 560 nm with corresponding emission peaks from 460 to 630 nm 30. Most studies used near-ultraviolet (330-380 nm) or blue light (450-490 nm) to excite green to yellow lipofuscin autofluorescence 25, 55, 56. But these light sources also excite autofluorescence from NAD(P)H and FAD, which will interfere with the lipofuscin quantification. Here, with near-infrared two-photon excitation at 1040 nm, we could selectively excite the red-end autofluorescence of lipofuscin and avoid the co-excitation problems 57, 58. That's how we successfully identify the specific lipofuscin-like red autofluorescence in macrophages. It is also noteworthy that two-photon excitation of NAD(P)H and FAD also excite lipofuscin's green to yellow fluorescence in macrophages. So the calculated redox ratio is a blended contribution from glucose metabolism and lipofuscin accumulation in macrophages. However, the lipofuscin fluorescence has almost no effect on NAD(P)H lifetime because we used a 460 ± 25 nm bandpass filter to reject the lipofuscin fluorescence while collecting the NAD(P)H signal.

Mechanisms involved in lysosomal lipofuscin accumulation include aging 33, oxidative stress 25, or apoptosis 59. Phagocytosis of lipofuscin-loaded dead cells may lead to lipofuscin accumulation in macrophages 60. Since macrophages contain a lot of lysosomes for phagocytosis, they usually display lipofuscin-laden features in several organs, such as the liver 26, lymph node 61, brain 27, and bone marrow 32. As a result, our specific metabolic fluorescence imaging of lipofuscin could identify the resident macrophages in adipose tissues. Furthermore, we found that adipose tissue macrophages in diabetic mice had higher red fluorescence of lipofuscin pigments, especially in CLSs than that of control and prediabetic ones (**Figure 4A**). Obesity and diabetic microenvironment could trigger inflammaging and immunosenescence 62, 63, which may cause more lipofuscin deposition in macrophages. In addition, the accumulation of lipofuscin pigments could be a dangerous signal, driving the production of pro-inflammatory chemokines and cytokines, which in turn promotes the activation of macrophages 44-46. Substantial lipofuscin pigments have been found in CD68-positive macrophages 64. Therefore, it is reasonable to speculate that the pro-inflammatory macrophages may have more deposition of lipofuscin pigments and higher lipofuscin red autofluorescence intensity. However, the mechanism by which lipofuscin accumulation affects macrophage phenotypic switch remains unclear. The role of lipofuscin-laden macrophages in diabetic microenvironments requires further study.

The causal link between inflammation and IR is still a controversial issue in the etiology of diabetes mellitus 65. Some studies demonstrated that adipose tissue inflammation is a trigger of IR 66-68, while other studies asserted that inhibition of inflammation not only fails to improve IR but instead causes glucose intolerance 69-72. In our study, mice developed IR by the 5^th^-week post-HFD or HFHSD feeding, whereas adipose tissue inflammation was not detectable until 4 months of feeding, indicating that diet-induced IR precedes inflammation. Interestingly, there was no change in the inflammation-related genes' expression in prediabetic adipose tissues, but the expression of ETC-related genes was downregulated (**Figure 2K**). Previous studies have shown that mitochondrial dysfunction is a key driver of IR 73-75. Excessive dietary intake of high fat and high sucrose creates a microenvironment with high free fatty acids and glycemia, causing oxidative stress and mitochondrial dysfunction in adipose tissues. Consequently, damaged mitochondrial function lessens mitochondrial biogenesis and mtDNA concentrations and inhibits the oxidation process, which impairs adipogenesis, lipogenesis, and adiponectin production 76, 77. Collectively, this causes enhanced inflammation and IR response in adipose tissues. As a warehouse for lipid storage, once adipose tissue has IR, it would cause lipid overflow and promote IR in other organs, such as the liver and skeletal muscle 78. Therefore, monitoring mitochondrial metabolism in adipose tissue could help the diagnosis of prediabetes. Changes in metabolic autofluorescence could reflect metabolic shifts earlier than those in genetic and proteomic levels 79. That's why autofluorescence measurement could enable the early detection of various diseases 80 and ensure dynamic, label-free, and non-destructive observation of tissue metabolism 81.

Of further importance, optical metabolic imaging delineates the metabolic heterogeneity of tissues at a single-cell level. Conventional biochemical methods can only perform bulk analysis of the whole tissue 82. For instance, NAD(P)+ / NAD(P)H detection kit detected bulk changes of NAD(P)+ / [NAD(P)H + NAD(P)+] ratio within a pooled population of cells and cannot separate the redox ratio of different cell types (**Figure S10**). White adipose tissue consists of a variety of cell types, including adipocytes, preadipocytes, endotheliocyte, macrophages, and other immune cells, and they serve different roles in the development and progression of IR and diabetes 83. There remains a clear need for performing metabolic analysis at the cellular level. Optical redox ratios, NAD(P)H lifetimes, and lipofuscin-like pigments not only distinguish macrophages from adipocytes but also characterize their separate metabolism in the development of diabetes. In this study, we successfully dissected the differentiable features of adipocytes and macrophages in prediabetic and diabetic microenvironments using the optical metrics described above. Only adipocytes' metabolic fluorescence changes during the prediabetic stage (**Figure 2**). The differentiable features of metabolic fluorescence were absent before the onset of IR and after its remission (**Figure S11, Figure 5**), indicating that our optical indices can specifically indicate IR in prediabetes pathology.

According to a long-term cohort study, the onset of IR has been reported as early as 10 years before the symptomatic phase of T2DM, and metabolic features and cellular molecules in people with IR vary with time lapsing 5. Since abnormal physiology arises in the early stages of chronic diseases, it is feasible to apply molecular imaging tools to diagnose inchoate diseases without apparent symptoms 84, 85. At present, several imaging tools are applied to diagnose different adipose depots. CT has been used to quantify and score visceral adipose tissue content in T2DM patients 86. MRI can calculate the ratio of visceral fat / subcutaneous fat volume, a promising index related to cardiovascular disease in individuals with impaired fasting glucose 87. Although CT and MRI are acknowledged imaging modalities for measuring visceral adiposity, there are some limitations, including professional requirements, costly, radiological hazards in CT, and the time-consuming of MRI 88, 89. Additionally, these conventional diagnostic techniques cannot monitor pathophysiological microenvironments 90. TPFM and FLIM have been widely applied to monitor disease microenvironments, such as cancer 80, neurodegenerative disease 91, and liver disease 92. The label-free and non-destructive feature of metabolic imaging by TPFM and FLIM guarantees a simple, safe, and reproducible detection method.

## Conclusion

In conclusion, our study validated that metabolic TPFM and FLIM imaging of NAD(P)H, FAD, and lipofuscin is a valuable method to label-free identify prediabetic adipose tissues. The lipofuscin fluorescence can indicate the location and activation status of macrophages. The redox metabolism of adipocytes could be analyzed at the location lacking lipofuscin fluorescence. These metabolic features can help improve the diagnostic accuracy of prediabetes, in particular when hyperglycemia is not significant in the early stages of IR development. However, this research still has certain limitations that warrant further studies. First, as we previously discussed, the redox ratios of macrophages have a mixed contribution from NAD(P)H, FAD, and lipofuscin, which does not accurately reflect the redox state of macrophages. There is a need to develop a calculation method that can correct the macrophage redox ratio based on the lipofuscin signal, and this is the work we will explore in the future. Second, our observations are made in visceral adipose tissue. As subcutaneous adipose tissue may be easier to access in clinical applications, future work needs to measure metabolic fluorescence changes in subcutaneous adipose tissues. Third, in this study, we demonstrated that optical parameters can distinguish between metabolically healthy and metabolically unhealthy adipose tissues in prediabetic or diabetic microenvironments. To determine whether metabolic changes in adipose tissues can specifically diagnose prediabetes, further studies with different disease animal models are required. Finally, this study has not elucidated the relationship between insulin resistance pathways and metabolic fluorescence, which requires further study.

In the future, we will integrate TPF microscopy and FLIM with fiber-based *in vivo* cell imaging platforms (Cellvizio, Mauna Kea Technologies) or transdermal techniques like optical microneedles 93 for prediabetes diagnosis. By employing optical fibers or microneedles, low-invasive detection of adipose tissue metabolic fluorescence could be achieved, and the data can be quickly transmitted back to the analysis software. By utilizing machine learning algorithms for data training, it is possible to rapidly predict the probability of having diseases and achieve goals of low-invasive, time-saving, as well as *in vivo* label-free screening for the diagnosis of prediabetes.

## Materials and Methods

### Development of the T2DM animal model

C57BL/6 mice were obtained from the Animal Laboratory, Faculty of Health Sciences, University of Macau. Lyz2^GFP^ mice have been described 94 and were kindly provided by Prof. Ivan Dzhagalov (National Yang Ming Chiao Tung University, Taipei, Taiwan). C57BL/6J-c2J-LysM-eGFP mice were crossbred from homozygous C2J and Lyz2^GFP^ mice. C57BL/6J and C57BL/6J-c2J-LysM-eGFP male mice were maintained on a 12-hour light/dark cycle with free access to food. All animal experiments were approved by the Animal Research Ethics Committee of the University of Macau (Protocol ID: UMARE-037-2019).

We used a high-fat diet (HFD) with 60% kcal fat (Research diet D12492i, Research Diets Inc., New Brunswick, NJ, USA) and a high-fat-high-sucrose diet (HFHSD) with 58% kcal fat and sucrose (Research diet D12331i, Research Diets Inc.) feeding protocols to induce the T2DM animal model. At the age of 6-7 weeks, mice were divided into three groups, including the control (fed with normal chow), HFD, and HFHSD groups, with 8 mice per group. Mice were fed the normal chow, HFD, or HFHSD for 4 months. We measured the mice's body weight, random blood glucose, and fasting glucose levels every week. Fasting insulin content was detected using Insulin Mouse ELISA Kit (EMINS, Invitrogen, Thermo Fisher Scientific). Homeostasis model assessment-IR (HOMA-IR) is used to evaluate the IR of individuals. The calculation method is as follows: fasting blood glucose level (FPG, mmol/L) × fasting insulin level (FINS, μU/mL)/22.5 95.

### Insulin tolerance test

Mice were fasted for 5 hours and injected intraperitoneally with 0.75 U/kg (bodyweight) of insulin solution (I9278, Sigma-Aldrich). Blood glucose concentration was detected with Roche's Blood glucose meter before insulin injection and at 15, 30, 45, and 60 minutes after injection.

### Intraperitoneal glucose tolerance test

After fasting for 12 hours, mice were weighed and injected with glucose solution at a dosage of 1 mg/g body weight. Blood glucose level was measured with a Roche Blood glucose meter before insulin injection and at 15, 30, 45, 60, 90, and 120 minutes after injection.

### Isolation and polarization of bone marrow-derived macrophages (BMDMs)

Bone marrow-derived macrophages were extracted from 6-8 weeks old age of C57BL/6J mice 96. Six mice were sacrificed for BMDMs' isolation and polarization. Mice were euthanized by cervical dislocation, and the femur and tibia bones were isolated. After rinsing off hair, bone joints were cut off, and marrow was flushed out using a 25 G needle into the 1640 medium containing 10% heat-inactivated fetal bovine serum (FBS) and 1% penicillin-streptomycin (PS). Then, cells were filtered, centrifuged, and resuspended in the 1640 medium containing 10% heat-inactivated FBS, 1% PS, and 10 ng/mL M-CSF (315-02, PeproTech), seeded into Petri dishes, and maintained in a humidified incubator with 5% CO_2_ at 37°C. On day 3, we changed the fresh BMDM growth medium, and on day 5, we seeded cells into different dishes. For M1 activation, we used 10 ng/mL LPS (L4391, Sigma) plus 20 ng/mL IFNγ (315-05, PeproTech). For M2 polarization, 20 ng/mL IL-4 (214-14, PeproTech) was used. Flow cytometry was applied to identify macrophage phenotypes.

### Flow cytometry assay

Cells were harvested, resuspended in PBS buffer containing 2% FBS, and filtered with a 0.22 µm strainer, followed by fluorescent-dye conjugated antibodies incubation at 37℃ for 30 minutes. After incubation, cells were analyzed by using BD Accuri™ C6 Plus Flow Cytometer (BD Biosciences). Antibodies used for macrophage phenotypes identification include PE-CD86 (105007, Biolegend, 1:20), PE-CD206 (141705, Biolegend, 1:40), PE-CD163 (12-1631-82, Biolegend, 1:80) and Alexa Fluor 647anti-mouse F4/80 antibody (123122, Biolegend, 1:200). CD86 is an M1 macrophage marker, while CD206 and CD163 are considered to be M2 macrophage markers 97-99.

### Whole-mount immunostaining

We followed a previously reported protocol 100. Fresh adipose tissues were fixed with 4% paraformaldehyde (PFA) overnight at room temperature. Fixed tissues were cut into 2-3 mm^3^ pieces, blocked with 5% BSA for 1 hour at room temperature, and incubated with FITC anti-mouse F4/80 Antibody (123107, Biolegend, 1:100) or Alexa Fluor 647 anti-mouse F4/80 antibody (123122, Biolegend, 1:100) or Anti-prosaposin (PSAP) antibody (ab180751, Abcam, 1:100) at 4ºC overnight, followed by Cy2 conjugated secondary antibody (ab6940, Abcam, 1:500) at room temperature for 1 hour. Observation of treated fat tissues was performed on TPFM. F4/80 is a major marker of macrophages 101, and prosaposin (PSAP) is a marker of lipofuscin 102, 103. Both F4/80 and PSAP are used to label macrophages.

### Co-localization of lysosome and lipofuscin fluorophore

BMDMs or freshly isolated adipose tissue was stained with 100 nM lysotracker green (L7526, Thermo Fisher Scientific., Waltham, MA, USA) for 30 minutes at 37 ℃. Lysosomes and lipofuscin-like fluorophores were visualized in TPFM under the excitation wavelength of 960 nm and 1040 nm, respectively. Co-localization of green fluorescence emitted by lysotracker green and lipofuscin-like red autofluorescence was analyzed by calculating Pearson's correlation coefficient (PCC) and Manders' Co-localization Coefficients (MCC).

### The TPFM and redox ratio analysis of NAD(P)H and FAD in freshly excised visceral fat

Fresh adipose tissue was excised and maintained in a 37°C micro-incubator (CU-501, Live Cell Instrument Co., LTD, Korea) containing 5% CO_2_ during imaging. For TPF imaging acquisition, we used a multiphoton microscopy system excited by a wavelength-tunable (700-1300 nm) femtosecond laser (InSight X3, Spectra-Physics, Newport). In each batch of measurement, we ensured the average excitation power after 20× NA = 0.75 objective is about 25 mW. The third harmonic generation (THG) intensity at the air-glass interface was used to calibrate laser excitation intensities. To mitigate the effect of imaging depth on excitation efficiency, we acquired Z-stack images of freshly excised adipose tissue within a depth of 20 μm and a step size of 2 μm, using a Nikon Inverted Microscope (A1R MP+/Ti2-E, Nikon instrument Inc.) with a 20× NA = 0.75 objective (Nikon CFI Plan Apo λ 20×/0.75 DIC N2, Nikon instrument Inc.). The sizes of images in pixels and physical dimensions are 4096×4096 pixels and 655.36×655.36 μm, respectively.

Excited at 890 nm, 740 nm, and 1040 nm, the TPF signals of FAD, NAD(P)H, and lipofuscin fluorescence can be generated, respectively. The TPF signal of FAD was detected by a channel with a 510-600 nm detection range. The detection range of the channel for NAD(P)H TPF signal was 400-480 nm 81. And the TPF signal of lipofuscin-like fluorescence was collected in a range of 604-679 nm 30. We took three Z-stack images for each adipose tissue to ensure that at least 30 adipocytes or 30 macrophages could be analyzed. We used the Image J software to process a sum of intensity projection of a Z-stack image and obtain the summed NAD(P)H or FAD fluorescence intensity (photon counts) in each pixel. Then, the redox ratio within each cell can be calculated using a formula of I_FAD_ / [I_NAD(P)H_ + I_FAD_] 81, where I_NAD(P)H_ and I_FAD_ are fluorescence intensities of NAD(P)H and FAD within a cell. The measurement and calculation of BMDM's redox ratio were the same as above.

### The FLIM and fluorescence lifetime of NAD(P)H in freshly excised visceral fat

Measurement of fluorescence lifetime in fresh epididymal fat or BMDMs was conducted on the Nikon Inverted Research Microscope (A1R MP+/Ti2-E, Nikon instrument Inc.) equipped with a femtosecond laser tunable between 700-1300 nm. Fresh adipose tissues or BMDMs were maintained in the micro-incubator system during FLIM image acquisition.

We used 740 nm femtosecond pulses to excite the NAD(P)H two-photon fluorescence and the fluorescence signals were collected by a 460 ± 25 nm bandpass filter (ET460/50m, Chroma Technology). The average excitation power after the 40× NA=1.15 objective (Nikon CFI Apo LWD λS 40×/1.15 WI, Nikon instrument Inc.) is about 10 mW. We tried to image different tissues at almost the same depth beneath the surface. First, we searched the outermost layer of adipose tissues, where there is a strong second harmonic generation (SHG) signal of collagens. Then, we further moved the imaging plane 20 μm deeper into the tissue to take the FLIM images. The FLIM data were detected by a high-speed photomultiplier tube (DCC-100, Becker & Hickl GmbH) and recorded by the time-correlated single-photon counting (TCSPC) module (SPC-160, Becker & Hickl GmbH). As confirmed with the FLIM of urea crystal, the instrument response function (IRF) has a 170 ps full-width half maximum. Each 256×256 pixels FLIM image was acquired within 120 sec to avoid photobleaching. To collect photon counts of more than 200 in most of the pixels, we set a 25.21 μs pixel dwell time. For NAD(P)H FLIM data analysis, we used an IRF convoluted two-component model 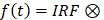
(a_1_e^-t/τ1^ +a_2_e^-t/τ2^) to fit the decay traces, where t_1_ and t_2_ represent short and long-lifetime components, and a_1_ and a_2_ are relative fraction of the lifetime components (a_1_ + a_2_ = 1) 104. The t_1_ and a_1_ are characteristics related to free NAD(P)H, but t_2_ and a_2_ are protein-bound NAD(P)H.

### Assessment of 2-NBDG glucose uptake in adipose tissues

Adipose tissues' glucose uptake function was measured using a Cell Meter™ 2-NBDG Glucose Uptake Assay Kit (36702, AAT Bioquest). Fresh adipose tissue was incubated with 0.5 mM 2-NBDG for 1 hour in a 37°C, 5% CO_2_ incubator. The fluorescence signal of 2-NBDG (Ex. @ 488 nm/Em. @ 530 nm) was monitored using the Nikon Inverted Research Microscope with a 20× 0.75 NA objective. We captured at least three Z-stack images of each adipose tissue (~20 μm depth of field, 2 μm step size), and used the Image J software to perform maximum intensity projection of Z-stack images to quantify the fluorescence intensity of each image.

### Biochemical measurement of NAD(P)+/NAD(P)H redox ratio

A tissue sample (about 10 mg) was cut into pieces with scissors and placed in a homogenizer with 400 μL of NAD+/NADH or NADP+/NADPH extraction solution. The tissue was homogenized on ice and centrifuged at 12,000 g for 10 minutes at 4°C, and the supernatant was taken for detection. The total amounts of NAD+/NADH and NADP+/NADPH in samples were determined according to the assay kit's protocol (S0175 & S0179, Beyotime). Based on the absorbance value and NAD(P)H standard curve, the redox ratio of NAD(P)+ / [NAD(P)H + NAD(P)+] was calculated.

### Hematoxylin and Eosin (H&E) staining

Freshly excised adipose tissues were fixed with 4% PFA overnight at room temperature, followed by dehydration. Dehydration was carried out in a dehydrator with gradient alcohol in sequence. After replacing the alcohol in the tissue with xylene, it could be embedded in paraffin. 5-10 μm slices were cut on a microtome (Leica RM2235, Wetzlar). Dewaxed slices were stained in an aqueous hematoxylin solution for a few minutes, then rinsed in acid water and the ammonia water for serval seconds each, followed by 1 hour in running water. After dehydration in 70% and 90% alcohol, slices were stained in alcohol eosin solution for 2-3 minutes. The stained sections were dehydrated with pure alcohol and then made transparent with xylene. The clear sections were dripped with neutral gum and covered with a coverslip for further image acquisition in a slide image scanner (NanoZoomer S60, Hamamatsu). Regarding the counting of CLS numbers, we took three fields of view for each H&E section and counted the number of CLSs in each field.

### Immunohistochemistry (IHC) labeling of macrophage markers

Dewaxed slices were first incubated with 3% H_2_O_2_ for 10 minutes at room temperature (protected from light) to block endogenous peroxidase, then subjected to microwave antigen retrieval. Next, slices were blocked with 5% BSA for 15 minutes at 37℃ and incubated with primary antibodies overnight at 4℃, followed by incubation of secondary antibodies for 40 minutes at 37℃. Slices were incubated with Streptavidin-Biotin Complex (SABC-HRP Kit, Beyotime) for 40 minutes at 37℃ and developed color by using DAB (DAB-HRP Color Development Kit, Beyotime). Finally, the slides were counterstained with hematoxylin, dehydrated with graded alcohol, cleared with xylene, mounted with neutral gum and coverslips, and examined by microscopy. Primary antibodies used in this project include an Anti-F4/80 antibody [SP115] (ab111101), an Anti-CD68 antibody (ab125212), a CD80 Monoclonal Antibody (2A2) (MA5-15512), an Anti-iNOS (ab3523) and an Anti-Mannose Receptor (CD206, ab64693). Secondary antibodies include HRP Anti-Rabbit IgG antibody (ab288151). For the quantitative analysis of IHC-labeled images, we also took three fields of view for each IHC-labeled slice and quantified the mean optical density of each image using Image Pro Plus software. The difference in relative mean optical density reflects the difference in relative protein expression between groups.

### RNA Sequencing and bioinformatic analysis

Mice epididymal fat tissues were frozen in liquid nitrogen immediately after isolation. Total RNA extraction and mRNA library were done by Novogene. The mRNA sequencing data were produced by Illumina Novaseq 6000. Raw data were cleaned by the Trim_glore software (version 0.6.2) and qualified filtered data was generated by FastQC software (version 0.11.8). Then reads were aligned to the mouse (Mus musculus) reference genome (GRCm38.p6 assembly version) by STAR software (version 2.7.0f) and gene counts were calculated by featueCounts software (version 1.6.4) separately. The raw count data were imported into R language (version 4.2.1) for further exploration.

The transcript per million (TPM) was calculated based on the sequencing depth and gene length, and normalization of TPM was used for principle component analysis (PCA) and heatmap visualization. For differential gene analysis, we use the R package DESeq2 (version 1.36.0) for comparison. The differential expression genes (DEGs) were defined based on the fold change, p.adj, and p-value. The thresholds are: fold change > 2, and false discovery rate (FDR) < 0.05. The gene ontology (GO) enrichment analysis was performed by the ClusterProfiler package (version 4.4.4).

### Seahorse assay

To analyze the bioenergetic profile of adipose tissue, we employed the XFe-24 Extracellular Flux Analyzer (Seahorse Bioscience) 105. For the Mito stress test, freshly excised adipose tissues were rinsed with assay medium (Seahorse XF DMEM Medium, 103575-100, Agilent Technologies) containing 1 mM pyruvate, 2 mM glutamine, and 10 mM glucose at pH 7.4. Approximately 9 mg of fat tissue was weighed and placed into the XF24 Islet Capture Microplate (101122-100, Agilent Technologies). Then, 500 μL of assay medium was added to each well of the XF24 Islet Capture Microplate and the tissue samples were incubated at 37 ℃ without CO_2_ for 45 minutes. The oxygen consumption rate (OCR) was measured after sequential injection of a set of cell stress test compounds, including 30 μM oligomycin, 2 μM fluoro-carbonyl cyanide phenyl-hydrazone (FCCP), and 5 μM rotenone plus 5 μM antimycin (Rot/AA). Basal respiration was calculated by subtracting the OCR after Rot/AA addition from the initial OCR. ATP production was determined by the difference between initial OCR and OCR after oligomycin injection. The maximum respiration was estimated as the difference between the OCR after FCCP injection and the OCR after Rot/AA addition.

For the glycolysis stress test, the assay medium was supplemented with 2 mM glutamine. The extracellular acidification rate (ECAR) was detected after the sequential addition of glycolysis stress test reagents, including 30 mM glucose, 5 μM oligomycin, and 100 mM 2-DG. Glycolysis was presented by the ECAR rate after glucose injection. Glycolytic capacity was defined as the ECAR rate after oligomycin addition. The glycolytic reserve was calculated as the ECAR after oligomycin injection minus ECAR after glucose addition.

### Statistical analysis

For the colocalization analysis on the images extracted from Image J software, we used Pearson's correlation coefficient (PCC) and Manders' Co-localization Coefficients (MCC) 106.

The binary classification accuracies of various optical metrics were calculated using logistic regression and the leave-one-out cross-validation method in SPSS 26.0 software program (SPSS Inc., USA). The metrics include the redox ratio, NAD(P)H fluorescence lifetime fitting parameters a_1_, t_1_, t_2_, and lipofuscin fluorescence intensity. We used a binomial logistic regression model in SPSS software to calculate the probability of disease prediction. First, we performed a univariate logistic regression analysis to screen for variables that have a statistically significant association with prediabetes or diabetes. The forest plot, which was generated using GraphPad Prism 9.0 software, shows the 95% confidence interval (CI) of the odds ratio (OR) and the *P* value of the null hypothesis (see Supplementary Materials). Next, we built a binomial logistic regression model with the screened variables to predict the probability of disease using SPSS software. For every single index or combined indices, we plotted their ROC curves and calculated the AUC to evaluate their performance in distinguishing the healthy, prediabetic, and diabetic status of mice. The ROC curve is also used to find out optimal diagnosis thresholds, sensitivity, and specificity for each index.

For plotting the Z-score heatmap, we calculated the mean and standard deviation of 9 optical readouts (ORs) for the control group (**Table S4**), which included adipocyte's redox ratio (OR1), adipocyte's NAD(P)H fluorescence lifetime fitting parameters -a_1_, t_1_, t_2_ (OR2-4), macrophage's redox ratio (OR5), macrophage's NAD(P)H fluorescence lifetime fitting parameters -a_1_, t_1_, t_2_ (OR6-8) and macrophage's lipofuscin fluorescence intensity (OR9). The Z-scores of optical readouts in the control, prediabetes, and diabetes groups were calculated using SPSS software. We then computed the average Z-scores of different optical readouts in each group (**Table S4**) and converted them into heatmaps using GraphPad Prism 9.0 software.

To integrate these optical readouts for the differential diagnosis of diabetes stages, we performed the principal component analysis (PCA). The PCA analysis treats each optical readout as an independent dimension and finds the two optimal eigenvectors PC1 and PC2, along which the data points between groups could be best separated. We performed PCA analysis using R software (version 3.1.1) and the omic-share online tool (https://www.omicshare.com/tools/Home/Soft/getsoft/type/index). Results show that PC1 and PC2 vectors are:

PC1 = -0.41 × OR1 + 0.36 × OR2 + 0.16 × OR3 - 0.33 × OR4 - 0.32 × OR5 + 0.32 × OR6 + 0.21 × OR7 + 0.40 × OR8 + 0.39 × OR9

PC2 = 0.074 × OR1 - 0.41 × OR2 + 0.53 × OR3 + 0.57 × OR4 + 0.015 × OR5 + 0.15 × OR6 + 0.26 × OR7 + 0.31 × OR8 + 0.15 × OR9

in HFD and:

PC1 = -0.36 × OR1 + 0.33 × OR2 + 0.019 × OR3 - 0.37 × OR4 - 0.25 × OR5 + 0.37 × OR6 + 0.30 × OR7 + 0.47 × OR8 + 0.34 × OR9

PC2 = 0.058 × OR1 - 0.094 × OR2 + 0.73 × OR3 + 0.43 × OR4 + 0.12 × OR5 + 0.30 × OR6 - 0.12 × OR7 + 0.042 × OR8 + 0.39 × OR9

in HFHSD.

They contribute 49.3% and 48.2% proportion of variance, respectively.

Data were shown as mean ± SD. GraphPad Prism 9.0 software was used for statistical analysis. An unpaired Student's t-test and one-way ANOVA with Tukey's post hoc test was performed to determine the significant differences between two groups and among three groups, respectively. The significant level for hypothesis testing is chosen as 0.05.

## Supplementary Material

Supplementary figures and tables, plots.

## Abbreviations

T2DM: type 2 diabetes mellitus
IR: insulin resistanc
CLSs: crown-like structures
PET/CT: positron emission tomography-computed tomography
MRI: magnetic resonance imaging
NAD(P)H: reduced nicotinamide adenine dinucleotide (phosphate)
FAD: oxidized flavin adenine dinucleotide
TPFM: two-photon fluorescence microscopy
FLIM: fluorescence lifetime imaging microscopy
ROC: receiver operating characteristic
AUC: area under ROC curve
OXPHOS: oxidative phosphorylation
ETC: electron transport chain
BMDMs: bone marrow-derived macrophages
ILs: interleukins
HFD: high-fat diet
HFHSD: high-fat-high-sucrose diet
HOMA-IR: Homeostasis model assessment-IR
FPG: fasting blood glucose level
FINS: fasting insulin level
FBS: fetal bovine serum
PS: penicillin-streptomycin
PCC: Pearson's correlation coefficient
MCC: Manders' Co-localization Coefficients
SHG: second harmonic generation
TCSPC: time-correlated single-photon counting
IRF: instrument response function
TPM: transcript per million
PCA: principle component analysis
DEGs: differential expression genes
FDR: false discovery rate
GO: gene ontology
OCR: oxygen consumption rate
ECAR: extracellular acidification rate
PSAP: prosaposin
FCCP: fluoro-carbonyl cyanide phenyl-hydrazone
Rot/AA: rotenone plus antimycin
THG: third harmonic generation
OR: odds ratio
CI: confidence interval
PCA: principal component analysis
