# Supplementary material for: Label-free optical metabolic imaging of adipose tissues for prediabetes diagnosis
Source: Theranostics. 2023 Jun 19;13(11):3550–67. doi: 10.7150/thno.82697 (PMC10334843; doi:10.7150/thno.82697)
Supplement: Supplementary file 1 — Supplementary figures and tables, plots. [file thnov13p3550s1.pdf]

Supplementary Materials

Label-free optical metabolic imaging of adipose tissues for prediabetes diagnosis

Liping Chen[1, 2], Guihui Qin[1, 2], Yuhong Liu[3], Moxin Li[1, 2], Yue Li[1, 2], Lun-Zhang Guo[4], Lidong Du[1, 2], Weiming Zheng[5], Pei-Chun Wu[1, 6], Yueh-Hsun Chuang[7], Xiaoyan Wang[1, 2*], Tzung-Dau Wang[8*], Ja-An Annie Ho[6*], Tzu-Ming Liu[1, 2*]

[1]Institute of Translational Medicine, Faculty of Health Sciences, University of Macau, Macao SAR, China.
[2]MOE Frontiers Science Center for Precision Oncology, University of Macau, Macao SAR, China.
[3]School of Pharmaceutical Sciences, Guangzhou University of Chinese Medicine, Guangzhou, 510006, China.
[4]Institute of Biomedical Engineering, National Taiwan University, Taipei 10617, Taiwan.
[5]Translational Medicine R&D Center, Zhuhai UM Science and Technology Research Institute, Zhuhai, China.
[6]Department of Biochemical Science & Technology, National Taiwan University, Taipei 10617, Taiwan.
[7]Department of Anesthesiology, National Taiwan University Hospital and College of Medicine, Taipei 10002, Taiwan.
[8]Cardiovascular Center and Division of Cardiology, Department of Internal Medicine, National Taiwan University Hospital and College of Medicine, Taipei 10002, Taiwan.

*Corresponding author. Tzu-Ming Liu, tmliu@um.edu.mo; Ja-An Annie Ho, jaho@ntu.edu.tw; Tzung-Dau Wang, tdwang@ntu.edu.tw; Xiaoyan Wang, xiaoyanwang85@yahoo.com. Address: Faculty of Health Sciences, University of Macau, Avenida da Universidade, Taipa, Macao SAR, China. TEL: +853-8822-4693.

Table of contents

Supplementary Text 3
Figure S1 7
Figure S2 9
Figure S3 11
Figure S4 12
Figure S5 13
Figure S6 15
Figure S7 16
Figure S8 18
Figure S9 18
Figure S10 20
Figure S11 20
Table S1. ROC analysis for adipocytes (1 month of feeding) 22
Table S2. ROC analysis for macrophages (4 months of feeding) 22
Table S3. ROC analysis for adipocytes (4 months of feeding) 23
Table S4. Mean and standard deviation (SD) of all optical readouts in control adipose tissues and the z-scores of all groups represented in the heatamps 23
Table S5. ROC analysis of PCA differentiation accuracy 25
Table S6. ROC analysis on optical deadouts of adipocytes for differentiating control from HFD and HFHSD-fed mice (feeding for 2 months) 25
Supplementary methods 27
Forest plot of Odds Ratios: Screening the association obetween optical matrics and diabetes 31
References 34

Supplementary Text

Validation that GFP-positive cells in adipose tissue of C57BL/6J-c2J-LysM-eGFP mice are macrophages.

An immunostaining assay was conducted to verify if GFP-positive cells in the epididymal fat of C57BL/6J-c2J-LysM-eGFP mice are macrophages. Fixed adipose tissue was stained with Alexa Fluor 647 anti-mouse F4/80 antibody. Under the excitation of 960 nm and 1200 nm respectively, GFP and Alexa Fluor 647 were visualized in adipose tissue (**Figure S1A**). GFP-positive cells can be labeled with Alexa Fluor 647 anti-mouse F4/80 antibody. GFP and Alexa Fluor 647 showed good co-localization, with a PCC value of 0.58 ± 0.042, an MCC-M1 value of 0.99, and an MCC-M2 value of 1. It suggests that GFP-positive cells express the F4/80 marker (**Figure S1B**).

In vitro validation of lipofuscin-like fluorescence as a label-free marker for macrophages.

Immunofluorescent staining of CD86, CD206, and CD163 with flow cytometric analysis confirmed that bone marrow-derived macrophages (BMDMs) were successfully polarized (**Figure S1C**). The characteristic fluorescence of lipofuscin-like pigments was almost absent in other cell lines, such as 3T3-L1 cells (**Figure S1D**). To further confirm that red autofluorescence is derived from lipofuscin-like pigments, co-localization analysis of lysosomal and lipofuscin-like fluorescence was performed in BMDMs (**Figure S1E**). Colocalization coefficient values including PCC (0.53 ± 0.048) and MCC (M1 = 0.93 ± 0.037, M2 = 0.97 ± 0.011) demonstrated a good correlation and high co-occurrence (**Figure S1F**). It can be determined that the fluorescent substance is a lipofuscin-like pigment according to the fluorescence emission spectrum (**Figure S1G**).

Exploration of prediabetes and diabetes models over time.

Previous studies have shown that in the presence of overnutrition, the metabolism of adipose tissue could be switched from OXPHOS to glycolysis, and adipose tissue macrophages are polarized to M1 macrophages. Besides, nutrient excess also affects macrophage mtDNA release, mtROS production, and mitochondrial dynamics and promotes obesity-induced inflammation [1-5]. Thus, in the present study, we chose the

dietary-induced diabetes model, which has unique advantages in simulating the etiology of human T2DM, especially the onset characteristics of obese patients with diabetes mellitus. Two different diets were used to induce diabetes models since pathologic responses may vary in different dietary components. Long-term exposure to a high-fat diet (HFD) with 60% kcal fat (D12492i, Research Diets Inc., New Brunswick, NJ, USA) causes several pathologies, including obesity, insulin resistance (IR) [6], inflammation [7], fatty liver [8], diabetic renal injury, and intestinal barrier dysfunction [9], and neurodegeneration [10]. Prolonged consumption of a high-fat-high-sucrose diet (HFHSD) with 58% kcal fat and sucrose (D12331i, Research Diets Inc., New Brunswick, NJ, USA) leads to obesity, IR, and diabetic myocardial damage [11], and fatty liver [12].

To find out when IR and diabetes developed in mice, we measured the mice's fasting blood glucose and insulin levels weekly. We found that the mice body weight of the HFD and HFHSD groups increased significantly compared with the control group fed with normal chow (**Figure S2A**). Although HFD- and HFHSD-fed mice exhibited elevated fasting insulin levels at weeks 5 and 6, respectively (**Figure S2B**), both groups had significantly higher HOMA-IR index at week 5 than the control group (**Figure 2A**). Mice fed HFD or HFHSD showed increased HOMA-IR value, suggesting the presence of IR [13, 14]. Prediabetes refers to an intermediate hyperglycemia [15, 16] state in which insulin resistance (IR) is already present and blood glucose levels are higher than normal but lower than those in diabetes. In this study, we expected to establish a prediabetes detection method, that is, the IR stage, so we chose the time around the onset of IR as the detection time point, the 4th week of HFD or HFHSD feeding. Comparing the metabolic profiles of prediabetes and diabetes is conducive to the establishment of detection technology for prediabetes, so it is also important to find out the onset of diabetes.

Although impaired fasting glucose levels were detected after 8 weeks of feeding on HFD or HFHSD (**Figure 2B**), these mice displayed clear-cut diabetes with fasting blood glucose levels of above 13 mmol/L at the 16th week, which was compatible with the previous study [17]. Furthermore, random blood glucose levels were significantly elevated only after 16 weeks of HFD or HFD feeding (**Figure S2C**). Thus, we set the 16th week of feeding on HFD or HFHSD as the time point for diabetes diagnosis. So, the period from the presence of IR until the fasting blood glucose level reaches 13 mmol/L is the prediabetic

state. That is, mice fed HFD or HFHSD for 4 to 15 weeks are considered prediabetic. From 16 weeks onwards, mice entered the diabetic stage. Besides, we found that IR precedes hyperglycemia, which is consistent with previous studies [18-20].

Anatomical diagrams of mice demonstrated that epididymal fat hypertrophy was observed in mice fed HFD or HFHSD for either 1 or 4 months (**Figure S2D-E**). Besides, we performed a 2-NBDG glucose uptake assay to verify that adipose tissue developed insulin resistance in mice fed HFD or HFHSD for 1 and 4 months. 2-NBDG is a fluorescent tracer used to monitor glucose uptake by living cells. Measurement of glucose uptake rate reflects insulin sensitivity [21, 22]. Fluorescence images and signals were acquired to ensure that 2-NBDG was absorbed by adipose tissue, and the result showed the peak of the spectrum was at 540 nm, which is the emission wavelength of 2-NBDG (**Figure S2F-G**). 2-NBDG uptake by epididymal fat was significantly reduced in mice fed HFD or HFHSD (**Figure S2H**), indicating that epididymal fat may have IR.

During the establishment of IR and diabetes mice models, in addition to tracking changes in fasting blood glucose and insulin levels, we also performed RNA-seq analysis of adipose tissue, looking for evidence at the transcriptomic level of whether the IR and diabetes mice models were successfully established. Heatmap clustering analysis manifested that HFD or HFHSD feeding for 1 month and 4 months resulted in the down-regulation of insulin receptor (Insr) and its substrate (Irs1) in adipose tissue, suggesting IR in adipose tissue. In addition, an increase of the adipokine leptin (Lep) was observed in the adipose tissue of mice fed HFD or HFHSD for 1 or 4 months (Supplementary Figure S2i, j). Up-regulation of leptin is associated with the increased risk of IR and T2DM [23-25]. Adiponectin (Adipoq) is another adipokine with the opposite biological function to leptin in inflammation and IR [26]. Compared with the control group, although the down-regulation of adiponectin and its receptor (Adipor2) was not detected at 1 month of HFD or HFHSD feeding, the expression of these two genes was down-regulated in both HFD and HFHSD groups after 4 months of feeding, indicating that long-term feeding of HF(HS)D causes more severe symptoms of adipose tissue IR than short-term feeding. Accumulated evidence revealed that mitochondrial dysfunction could be one of the pathogenic factors of IR and T2DM [27, 28]. Suppression of metabolic genes involved in mitochondrial biogenesis and glucose/fatty acid metabolism including proliferator

peroxisome-activated receptor (PPAR) coactivator-1α, PGC-1α (Ppargc1a), the PPARs members (Ppara, Ppard, and Pparg), and 5'AMP-activated protein kinase (AMPK) (Prkaa1, Prkaa2, Prkab2) were observed in T2DM subjects [29-31]. In our study, both short- and long-term exposure to HF(HS)D resulted in the down-regulation of these metabolic genes, implying mitochondrial dysfunction in adipose tissue. Evidence from RNA-seq supported the successful construction of IR and diabetes mice models in this study.

Figure S1

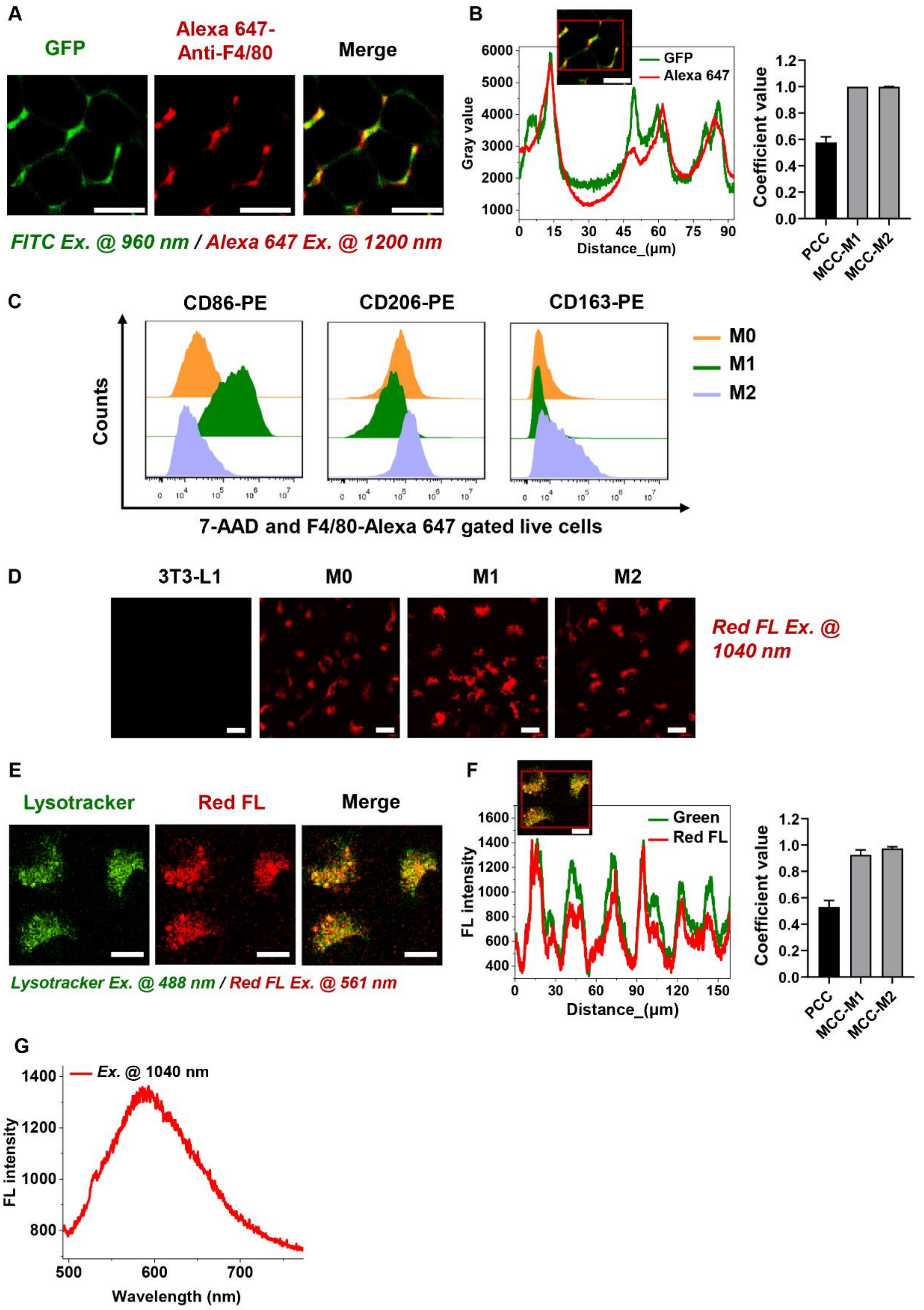

A
GFP
Alexa 647-Anti-F4/80
Merge
FITC Ex. @ 960 nm / Alexa 647 Ex. @ 1200 nm
B
GFP
Alexa 647
Gray value
Distance_(μm)
Coefficient value
PCC
MCC-M1
MCC-M2
C
CD86-PE
CD206-PE
CD163-PE
M0
M1
M2
Counts
7-AAD and F4/80-Alexa 647 gated live cells
D
3T3-L1
M0
M1
M2
Red FL Ex. @ 1040 nm
E
Lysotracker
Red FL
Merge
Lysotracker Ex. @ 488 nm / Red FL Ex. @ 561 nm
F
Green
Red FL
FL intensity
Distance_(μm)
Coefficient value
PCC
MCC-M1
MCC-M2
G
Ex. @ 1040 nm
FL intensity
Wavelength (nm)

Figure S1. Lipofuscin-like red autofluorescence is specific to adipose tissue macrophages. (A) Colocalization of GFP and macrophage marker F4/80 in the visceral fat of C57BL/6J-c2J-LysM-eGFP mice. GFP excited by 960 nm was presented by green color, and Alexa Fluor 647 anti-mouse F4/80 excited by 1200 nm was presented by red color. Scale bar: 50 μm. (B) Colocalization analysis for GFP and Alexa 647. PCC = 0.58 ± 0.04, M1 = 0.99, M2 = 1. (n = 3) (C) Flow cytometry assay for different macrophage phenotypes identification. (D) Representative images of lipofuscin-like red autofluorescence in 3T3-L1 cells and BMDMs (n = 6). Scale bar: 20 μm. (E) Representative images displaying colocalization of lysotracker green and lipofuscin-like red autofluorescence in BMDMs. Scale bar: 10 μm. (F) Colocalization analysis for lysotracker green and lipofuscin-like red autofluorescence. PCC = 0.53 ± 0.048, MCC-M1 = 0.93 ± 0.038, MCC-M2 = 0.97 ± 0.011. (G) The fluorescence emission spectra of lipofuscin-like pigments in macrophages. Data are presented as Mean ± SD (n = 3 or 6).

Figure S2

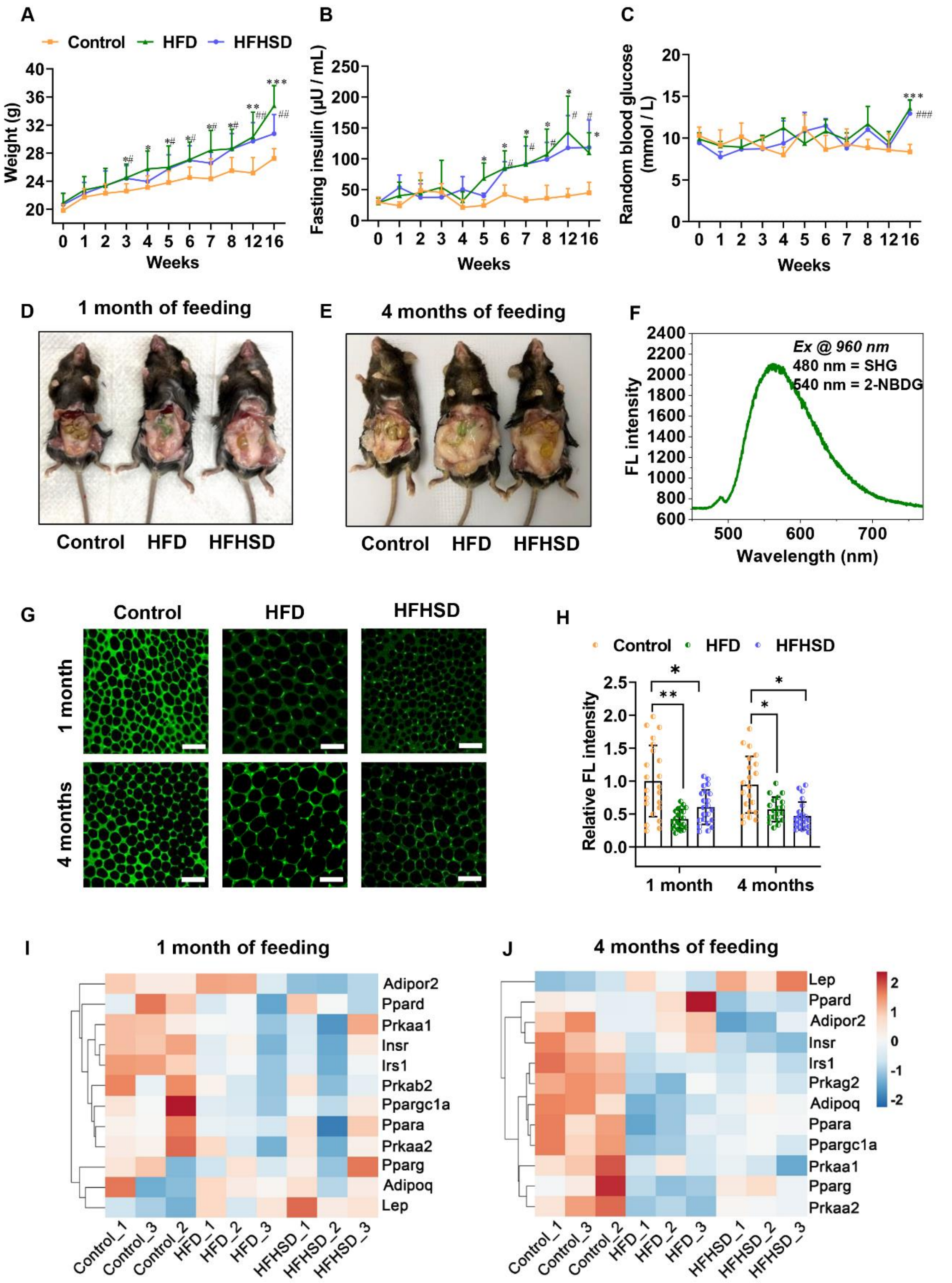

Figure S2. Establishment of Type II Diabetes Mellitus (T2DM)/Pre-T2DM model in mice. (A) Weight gain in mice fed HFD or HFHSD for 16 weeks. (B) Fasting insulin levels in mice fed HFD or

HFHSD for 16 weeks. (C) Random blood glucose levels in mice fed HFD or HFHSD for 16 weeks. (D-E) The representative anatomy diagram of mice (D: 1 month of feeding; E: 4 months of feeding). (F) The fluorescence emission spectra of 2-NBDG in adipose tissue. (G) Representative fluorescence images of 2-NBDG glucose uptake in fresh epididymal fat (Top: 1 month of feeding; bottom: 4 months of feeding). Scale bar: 100 μm. (H) Quantification of relative 2-NBDG fluorescence intensity in adipose tissue (Left: 1 month of feeding; right: 4 months of feeding). Each group contains 25-30 data points from 3 biological replicas. Each data point is the average of 10 cells in one field of view. (I-J) Heatmaps of expression profiles of insulin receptor genes, adipokine genes, and metabolic-related genes in adipose tissues. Data are expressed as Mean ± SD (n = 3). $^{*}P < 0.05$, $^{**}P < 0.01$, and $^{***}P < 0.001$, the HFD group *vs.* the control group; $^{\#}P < 0.05$, $^{\#\#}P < 0.01$, and $^{\#\#\#}P < 0.001$, the HFHSD group *vs.* the control group.

Figure S3

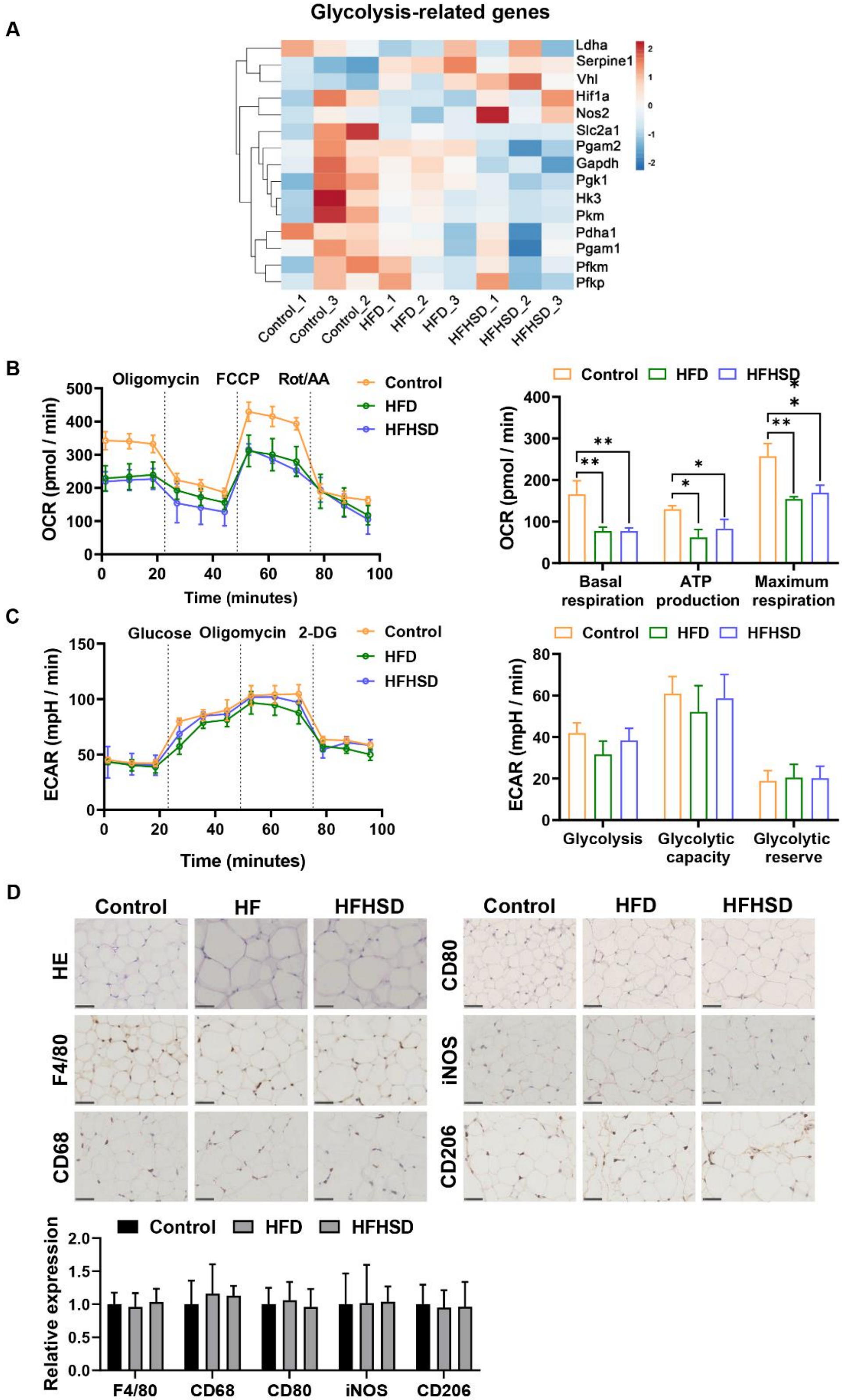
A
Glycolysis-related genes
Ldha
Serpine1
Vhl
Hif1a
Nos2
Slc2a1
Pgam2
Gapdh
Pgk1
Hk3
Pkm
Pdha1
Pgam1
Pfkm
Pfkp
Control_1
Control_3
Control_2
HFD_1
HFD_2
HFD_3
HFHSD_1
HFHSD_2
HFHSD_3
B
Oligomycin
FCCP
Rot/AA
Control
HFD
HFHSD
OCR (pmol / min)
Time (minutes)
Basal respiration
ATP production
Maximum respiration
C
Glucose
Oligomycin
2-DG
ECAR (mpH / min)
Glycolysis
Glycolytic capacity
Glycolytic reserve
D
Control
HF
HFHSD
HFD
HE
F4/80
CD68
CD80
iNOS
CD206
Relative expression

Figure S3. Identification of metabolic and inflammatory phenotypes of prediabetic adipose tissue. (A) Expression profile of glycolysis-related genes in adipose tissues of normal and prediabetic mice (HFD or HFHSD fed for 1 month). (B) (left panel) Seahorse analysis of oxygen consumption rate (OCR) of normal and prediabetic adipose tissues. (right panel) Comparative analysis of basal respiration, ATP production, and maximum respiration capacity in adipose tissues of healthy and prediabetic mice. (C) (left panel) Seahorse analysis of extracellular acidification rate (ECAR) of normal and prediabetic adipose tissues. (right panel) Comparative analysis of glycolysis, glycolytic capacity, and glycolytic reserve in adipose tissues of normal and prediabetic mice. (D) (upper panel) Hematoxylin and Eosin (H&E) staining and immunostaining of F4/80, CD68, CD80, iNOS and CD206 in epididymal fat from normal and prediabetic mice. (lower panel) Quantification of relative expression of F4/80, CD68, CD80, iNOS, and CD206 in adipose tissue paraffin sections. Data are expressed as Mean ± SD (n = 3). $^{*}P < 0.05$ and $^{**}P < 0.01$ relative to the control group.

Figure S4

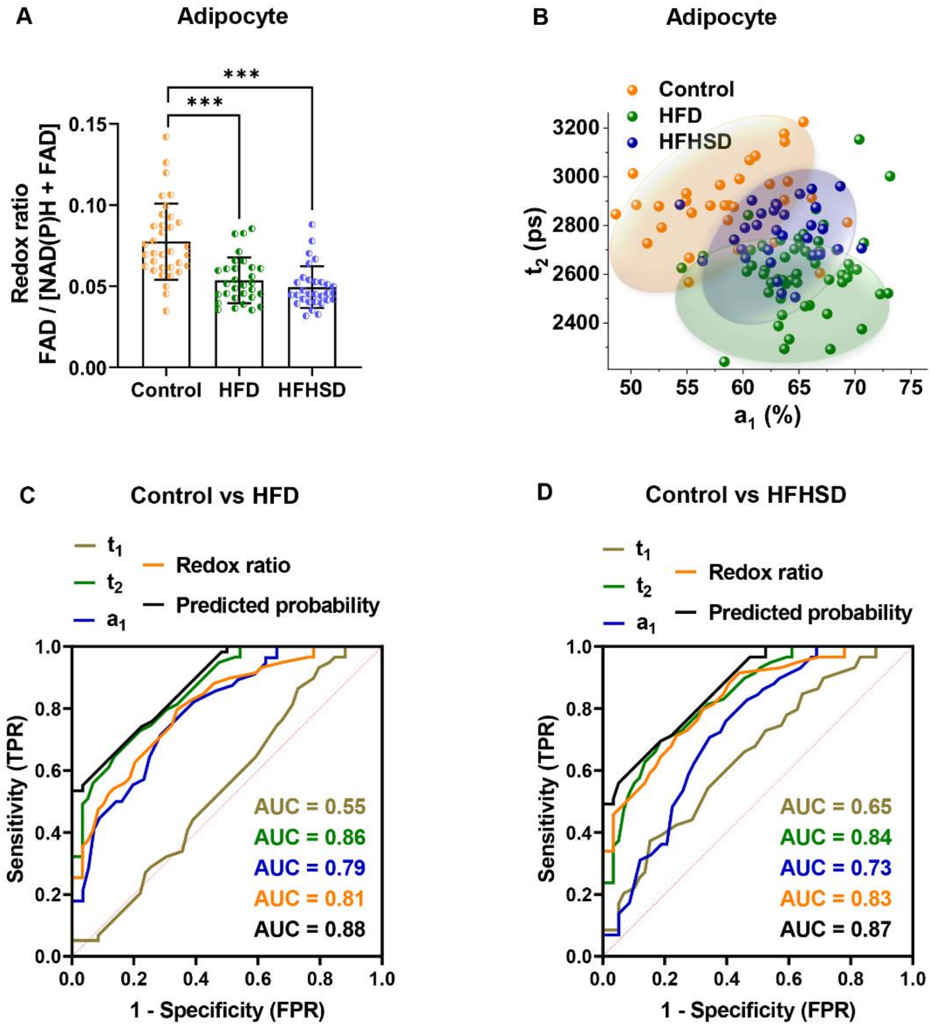

Figure S4. Optical metrics of adipocytes in diabetic adipose tissues. (A) Quantification of redox ratio in adipocyte cytoplasm of epididymal adipose tissues from mice fed HFD or HFHSD for 4 months. Data are presented as Mean ± SD. Each group contains 25-30 data points from 8 biological replicas. Each data point is the average of 10 cells in one field of view. $^{***}P < 0.001$ versus the control group. (B) The 2D scatter plot displayed that the two parameters a_1 and t_2 can distinguish the adipocytes of the control, HFD, and HFHSD groups, with an original classification accuracy of 91.2% (control versus HFD) or 85.5% (control versus HFHSD) and a cross-validation classification accuracy of 91.2% (control versus HFD) or 85.5% (control versus HFHSD). Each group contains 25-30 data points from 8 biological replicas. Each data point is the average of 10 cells in one field of view. (C, D) ROC curves and AUC values for optical readouts (redox ratio, a_1, t_1, t_2, and t_2-a_1-redox ratio-integrated parameter) of adipocytes, showing their ability to distinguish between (C) control and HFD groups or between (D) control and HFHSD groups. Other parameters of ROC and AUC analyses were shown in Table S3.

Figure S5

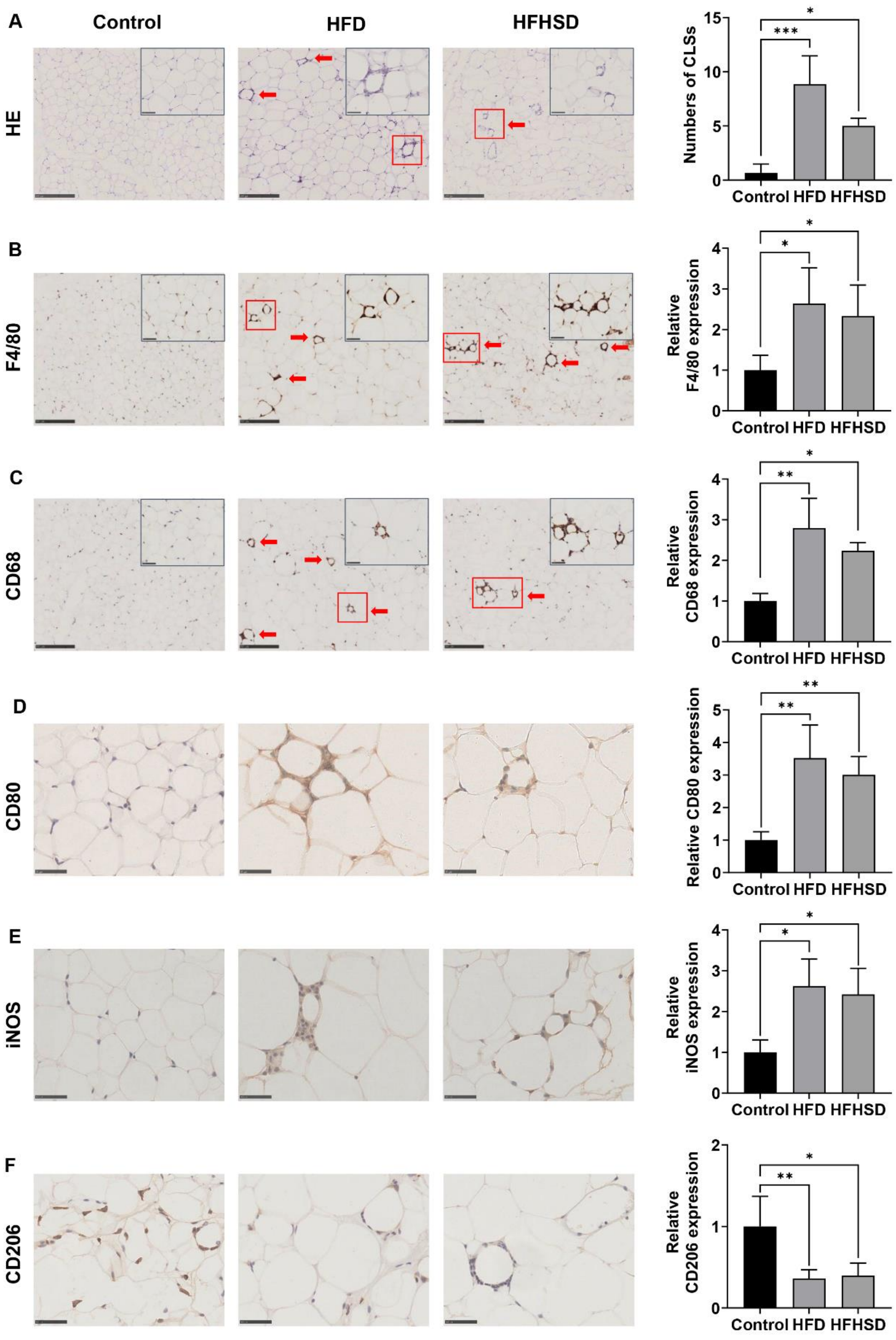

Figure S5. Histopathological examination of adipose tissues from mice fed for 4 months. (A) Hematoxylin and Eosin (H&E) staining of epididymal fat in control mice and diabetic mice (HFD and

HHFSD groups). The red arrow marks the crown-like structures (CLSs). The bar chart shows the number of CLSs. Immunostaining of (B) F4/80, (C) CD68, (D) CD80, (E) iNOS, and (F) CD206 in paraffin sections of adipose tissues showing CLSs. Relative expression of F4/80, CD68, CD80, iNOS, and CD206 in adipose tissue sections was quantified using Image Pro Plus. Scale bar: 250 μm for large images and 50 μm for inset images in (B) and (C), 50 μm in (D), (E), and (F). Data are presented as Mean ± SD (n = 3). $^{*}P < 0.05$, $^{**}P < 0.01$, and $^{***}P < 0.001$ versus the control group.

Figure S6

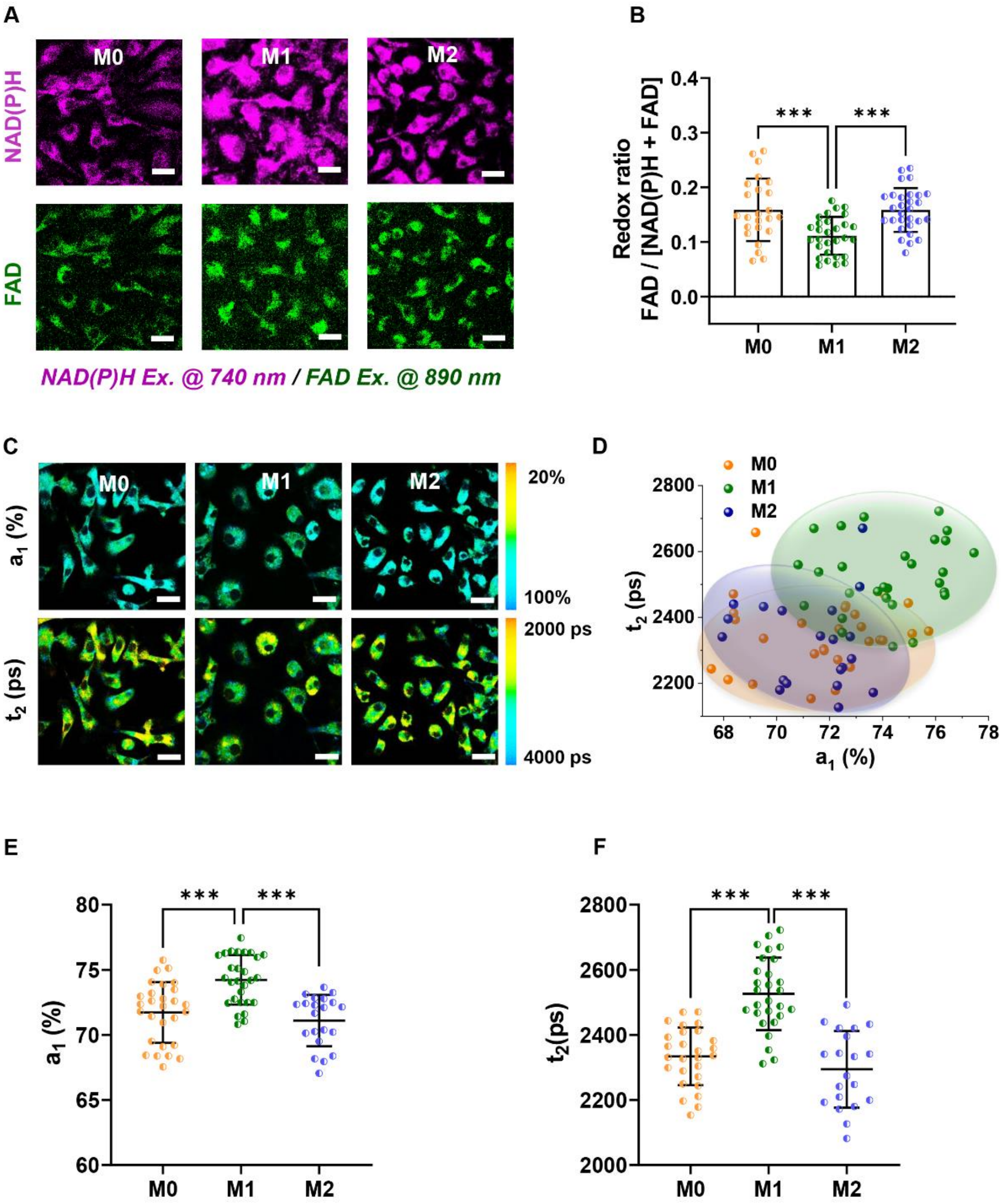

Figure S6. TPFM and FLIM metabolic imaging of BMDMs. (A) Representative images of NAD(P)H and FAD fluorescence intensity in different phenotypes of BMDMs, including M0, M1, and M2. Scale bar: 20 μm. (B) Quantification of redox ratio of M0, M1, and M2 macrophages. (C) Representative pseudo-color-coded FLIM a_1 and t_2 images of NAD(P)H of M0, M1, and M2 macrophages. Scale bar: 20 μm. (D) The a_1-t_2 scatter plot manifests a good separation of M1 from M0 (original classification accuracy: 83.9% cross-validation: 83.9%), and M1 from M2 (original classification accuracy: 87.8% cross-validation: 87.8%). Quantification of free NAD(P)H fraction a_1 (E) and long lifetime component t_2 (F) in BMDMs. Each group in (B, D, E, F) contains 25-30 data points from 6 biological replicas. Each data point is the average of 10 cells in one field of view. Data in (B, D, E, F) are presented as Mean ± SD. $^{***}P < 0.001$ compared with the control group.

Figure S7

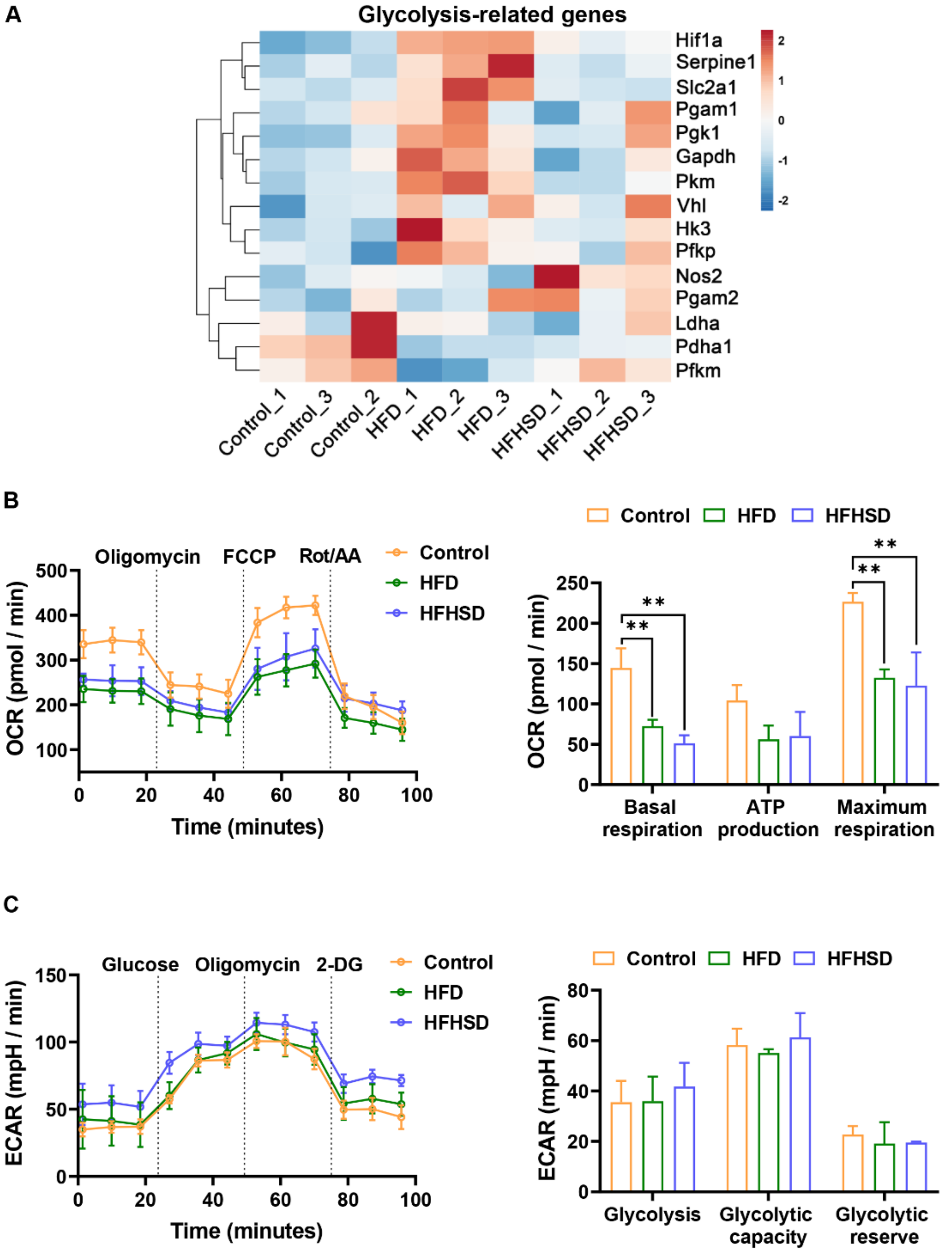

Figure S7. Identification of metabolic profile of diabetic adipose tissue. (A) Heatmap of glycolysis-related genes' expression in adipose tissues of the control and diabetic mice (HFD or HFHSD fed for 4 months). (B) (left panel) Oxygen consumption rate (OCR) profile plots of epididymal fat from the control and diabetic mice. (right panel) Quantification of basal respiration, ATP production, and maximum respiration capacity in epididymal fat of the control and diabetic mice. (C) (left panel) Extracellular acidification rate (ECAR) profile plots of adipose tissue from the control and diabetic mice. (right panel) Quantification of glycolysis, glycolytic capacity, and glycolytic reserve in adipose

tissues of the control and diabetic mice. Data are shown as Mean ± SD (n = 3). $^{**}P < 0.01$ relative to the control group.

Figure S8

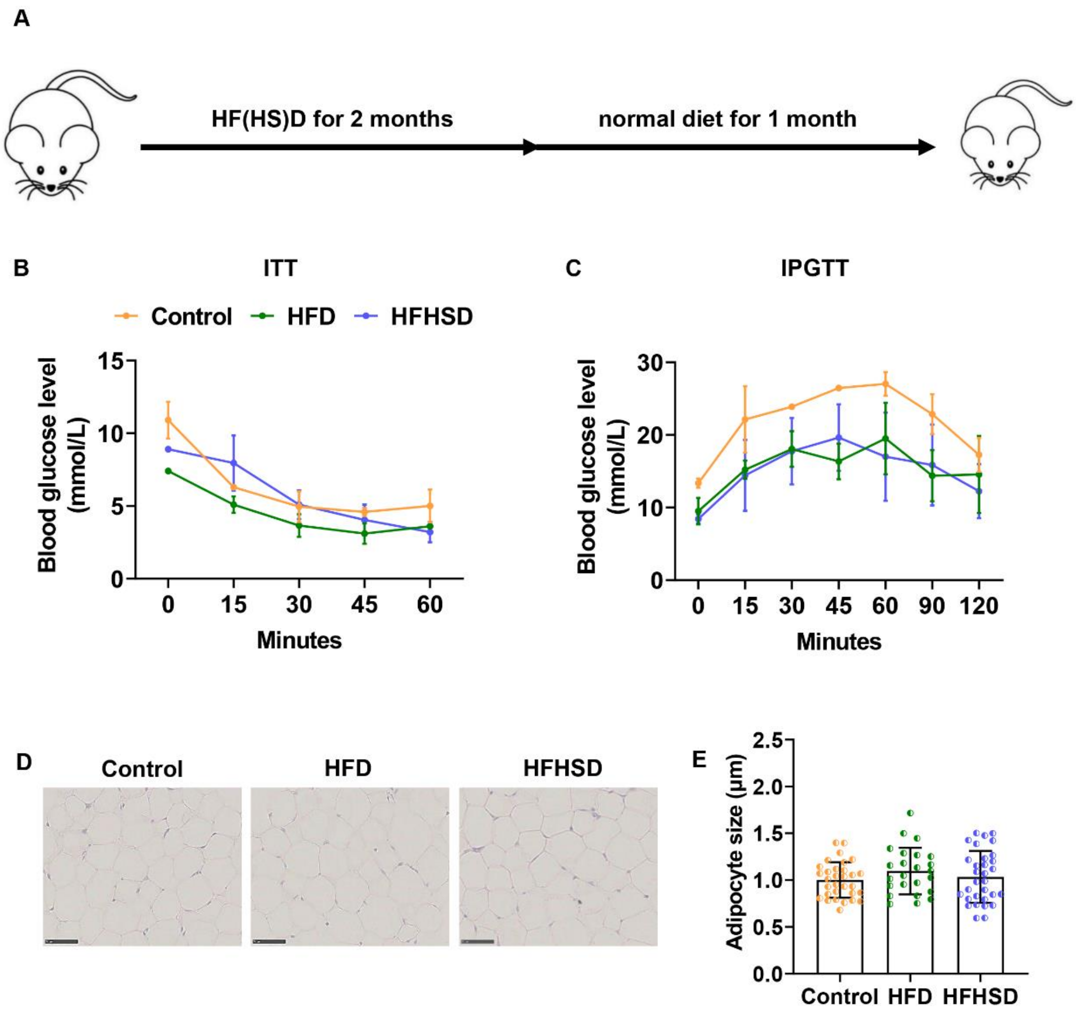

Figure S8. Diet changes improving prediabetes symptoms in mice. (A) Timeline showing mice fed HFD or HFHSD for 2 months, followed by normal chow for 1 month. Mice's blood glucose levels were monitored by using an insulin tolerance test (ITT) (B) and an intraperitoneal glucose tolerance test (IPGTT) (C) (n = 8). (D) HE staining of adipose tissue (n = 3). (E) Quantification of adipocyte size. Data are represented as Mean ± SD (n = 3 or 8).

Figure S9

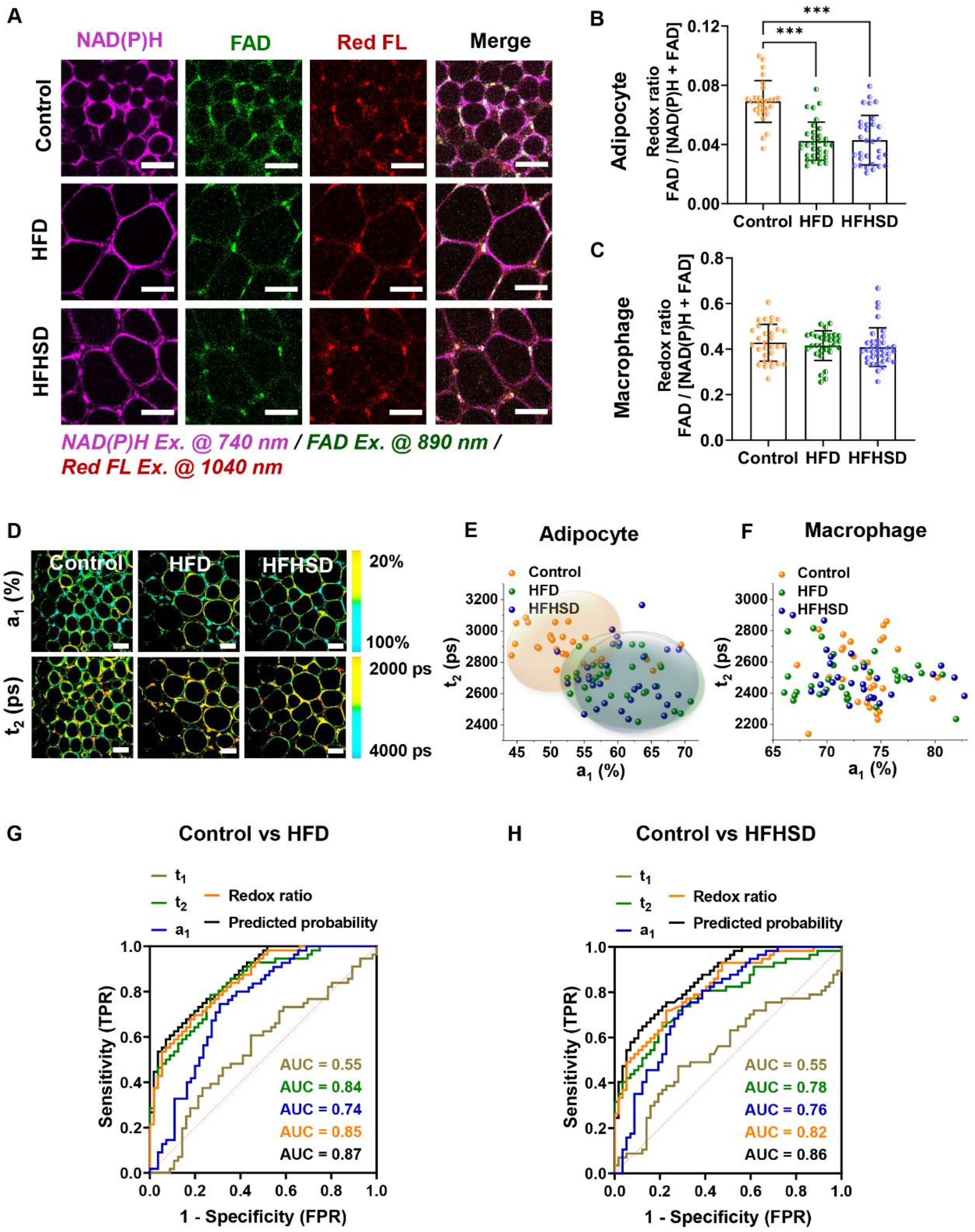

Figure S9. TPFM and FLIM metabolic imaging of epididymal fat in prediabetic mice. (A) Representative images of NAD(P)H, FAD, and lipofuscin-like fluorescence intensity in epididymal fat from mice fed HFD or HFHSD for 2 months. Scale bar: 50 μm. The redox ratios of adipocytes (B) and macrophages (C) were quantified. Data were presented as Mean ± SD (n = 8). $^{***}P < 0.001$ versus the control group. (D) False-color-coded FLIM a_1 and t_2 images of NAD(P)H in adipose tissues. Scale bar: 50 μm. The a_1-t_2 scatter plots of (E) adipocytes and (F) macrophages. (G, H) ROC curves and AUC

values for optical readouts (redox ratio, a_1, t_1, t_2, and t_2-a_1-redox ratio-integrated parameter) of adipocytes, showing their ability to distinguish between (G) control and HFD groups or between (H) control and HFHSD groups. Other parameters of ROC and AUC analysis were shown in **Table S6**. Each group in (B, C, E, and F) contains 25-30 data points from 8 biological replicas. Each data point is the average of 10 cells in one field of view.

Figure S10

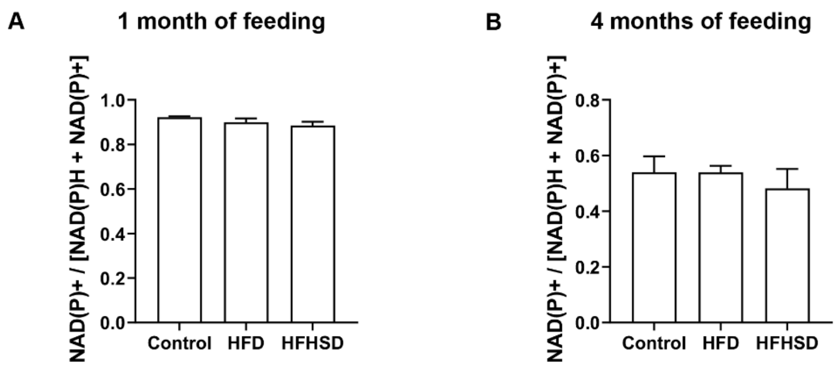

Figure S10. NAD(P)+/NAD(P)H content measurement. (A-B) Relative NAD(P)+/NAD(P)H content in adipose tissue. Adipose tissue from mice fed for 1 month (A) and 4 months (B) respectively. Data are represented as Mean ± SD (n = 3).

Figure S11

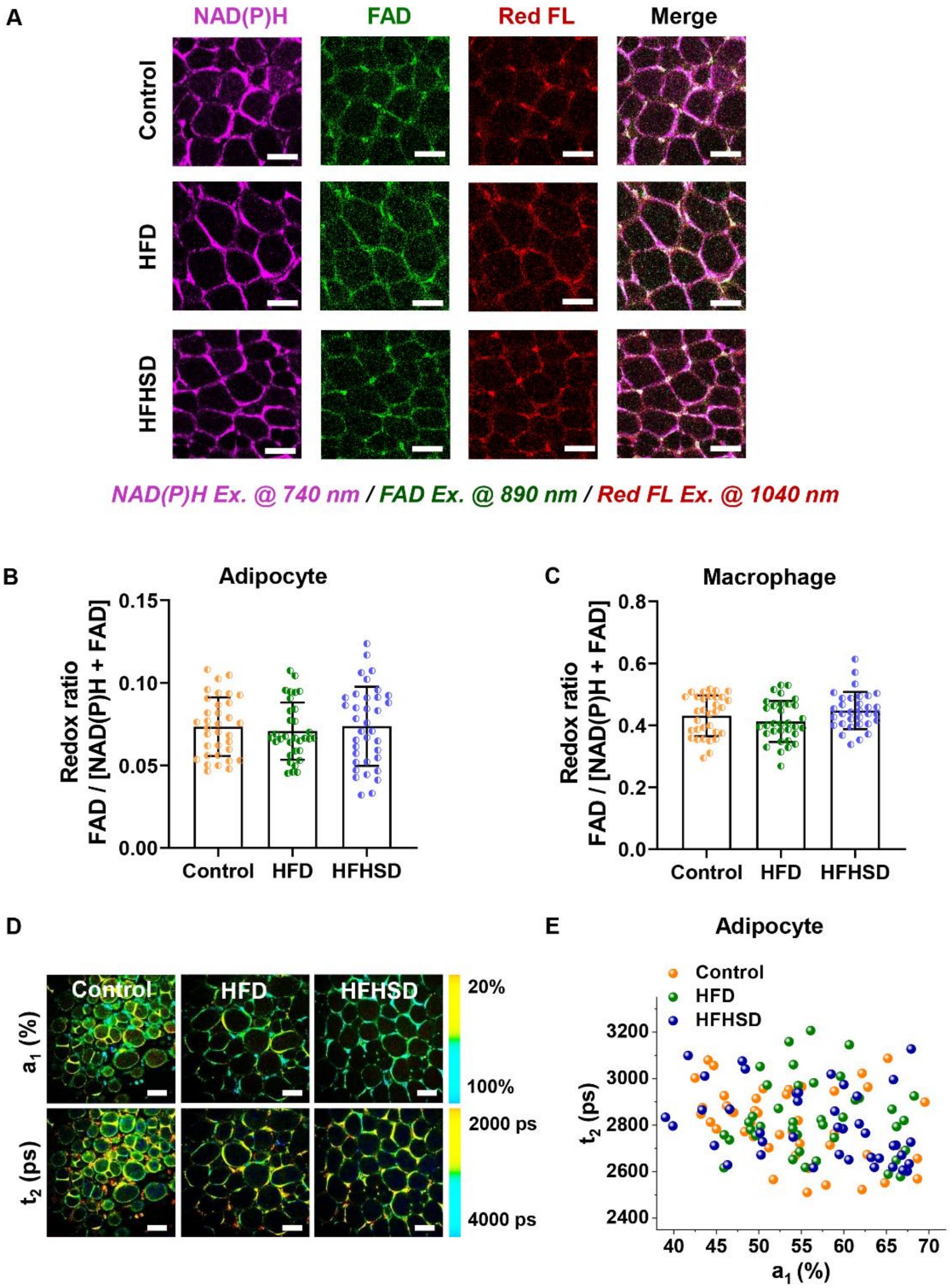

Figure S11. No changes in the redox ratio and NAD(P)H lifetime of adipocytes were observed in mice fed HFD or HFHSD for 2 weeks. (A) Representative images of NAD(P)H, FAD, and lipofuscin fluorescence intensity. Scale bar: 50 μm. (B) Quantification of redox ratio in adipocytes. (C) Quantification of redox ratio in macrophages. (D) FLIM images of adipose tissues. Scale bar: 50 μm. (E) The a_1-t_2 scatter plot. Data are represented as mean ± SD (n = 8). Each group in (B, C, E) contains 25-30 data points from 8 biological replicas. Each data point is the average of 10 cells in one field of view.

Table S1. ROC analysis for adipocytes (1 month of feeding)

		ROC curve				
Group	Optical index	Sensitivity	Specificity	AUC	Youden index	Cutoff
Control vs HFD	t_1	60.61%	50%	0.52	0.11	477
	t_2	93.94%	68.42%	0.82	0.62	2802
	a_1	93.94%	92.11%	0.87	0.86	60.05
	Redox ratio	90.91%	71.05%	0.8	0.62	0.08
	Predicted probability	96.97%	100%	0.96	0.97	0.58
Control vs HFHSD	t_1	35.14%	76.32%	0.5	0.11	507.6
	t_2	75.68%	84.21%	0.81	0.60	2729
	a_1	78.38%	92.11%	0.83	0.70	60
	Redox ratio	97.30%	68.42%	0.83	0.66	0.08
	Predicted probability	89.19%	100%	0.93	0.89	0.77

Table S2. ROC analysis for macrophages (4 months of feeding)

		ROC curve				
Group	Optical index	Sensitivity	Specificity	AUC	Youden index	Cutoff
Control vs HFD	t_1	88%	92.59%	0.87	0.81	451.1
	t_2	96%	92.59%	0.87	0.89	2345
	a_1	56%	81.48%	0.68	0.37	75.4
	Redox ratio	88%	62.96%	0.77	0.51	0.44
	Lipofuscin intensity	84%	81.48%	0.82	0.65	1.2
	Predicted probability	100%	96.30%	0.86	0.96	0.44

Control vs HFHSD	t_1	85.71%	100%	0.86	0.86	475
	t_2	91.43%	96.30%	0.87	0.88	2374
	a_1	57.14%	81.48%	0.71	0.39	75.39
	Redox ratio	77.14%	70.37%	0.76	0.48	0.43
	Lipofuscin intensity	60%	88.89%	0.74	0.49	1.37
	Predicted probability	94.29%	100%	0.88	0.94	0.63

Table S3. ROC analysis for adipocytes (4 months of feeding)

		ROC curve				
Group	Optical index	Sensitivity	Specificity	AUC	Youden index	Cutoff
Control vs HFD	t_1	96.67%	20%	0.55	0.17	609.9
	t_2	96.67%	86.67%	0.86	0.83	2778
	a_1	100%	56.67%	0.79	0.57	60.01
	Redox ratio	76.67%	83.33%	0.81	0.6	0.06
	Predicted probability	100%	96.67%	0.88	0.97	0.47
Control vs HFHSD	t_1	50%	76.67%	0.65	0.27	488.8
	t_2	86.67%	83.33%	0.84	0.7	2812
	a_1	96.67%	56.67%	0.73	0.53	59.7
	Redox ratio	83.33%	93.33%	0.83	0.77	0.056
	Predicted probability	100%	90%	0.87	0.9	0.30

Table S4. Mean and standard deviation (SD) of all optical readouts in control adipose tissues and the z-scores of all groups represented in the heatmaps

Statistics of optical readouts in the control group

Optical readouts	Mean	SD
OR1: Adipocyte_redox_ratio	0.090	0.022
OR2: Adipocyte_NAD(P)H_a_1 (%)	55.95	3.94
OR3: Adipocyte_NAD(P)H_t_1 (ps)	470	43
OR4: Adipocyte_NAD(P)H_t_2 (ps)	2827	120
OR5: Macrophage_redox_ratio	0.429	0.071
OR6: Macrophage_NAD(P)H_a_1 (%)	71.51	3.95
OR7: Macrophage_NAD(P)H_t_1 (ps)	470	60
OR8: Macrophage_NAD(P)H_t_2 (ps)	2510	151
OR9: Macrophage_lipofuscin_FL (Counts/cell)	10386	3864

Z-scores of all groups

Optical readouts	Control		Prediabetes		Diabetes	
	HFD	HFHSD	HFD	HFHSD	HFD	HFHSD
Adipocyte_redox_ratio	0.00	0.00	-1.16	-1.48	-1.71	-1.89
Adipocyte_NAD(P)H_a_1	0.00	0.00	2.73	2.14	2.90	2.27
Adipocyte_NAD(P)H_t_1	0.00	0.00	0.15	0.03	-0.03	-0.65
Adipocyte_NAD(P)H_t_2	0.00	0.00	-1.62	-1.35	-1.75	-1.62
Macrophage_redox_ratio	0.00	0.00	-0.05	-0.04	-1.21	-1.11
Macrophage_NAD(P)H_a_1	0.00	0.00	0.20	-0.04	0.95	1.26
Macrophage_NAD(P)H_t_1	0.00	0.00	-0.20	0.05	0.44	0.56
Macrophage_NAD(P)H_t_2	0.00	0.00	-0.52	-0.13	2.51	2.73
Macrophage_lipofuscin_FL	0.00	1.00	0.00	1.05	2.83	1.94

Table S5. ROC analysis of PCA differentiation accuracy

	Group	ROC curve				
		Sensitivity	Specificity	AUC	Youden index	Cutoff
**HFD-induced model**	Prediabetes vs Diabetes	100%	93.94%	0.99	0.94	0.31
	Control vs Prediabetes	96.97%	85.71%	0.85	0.83	0.40
	Control vs Diabetes	100%	97.14%	0.94	0.97	0.31
**HFHSD-induced model**	Prediabetes vs Diabetes	83.33%	100%	0.87	1	0.71
	Control vs Prediabetes	37.84%	97.14%	0.85	0.97	0.84
	Control vs Diabetes	73.33%	100%	0.88	1	0.98

Table S6. ROC analysis on optical readouts of adipocytes for differentiating control from HFD and HFHSD-fed mice (feeding for 2 months)

		**ROC curve**				
Group	Optical index	Sensitivity	Specificity	AUC	Youden index	Cutoff
Control vs HFD	t_1	68.97%	50%	0.55	0.19	559.6
	t_2	82.76%	92.86%	0.84	0.76	2782
	a_1	93.10%	60.71%	0.74	0.54	53.61
	Redox ratio	93.10%	89.29%	0.85	0.82	0.057
	Predicted probability	96.55%	92.86%	0.87	0.89	0.4
Control vs HFHSD	t_1	40%	82.14%	0.55	0.22	471.3
	t_2	70%	92.86%	0.78	0.63	2781
	a_1	86.67%	67.86%	0.76	0.55	55.36
	Redox ratio	83.33%	89.29%	0.82	0.73	0.057
	Predicted probability	93.33%	89.29%	0.86	0.83	0.43

Supplementary methods

Procedures for quantifying cellular redox ratio and NAD(P)H lifetime

Calculation of macrophage redox ratio

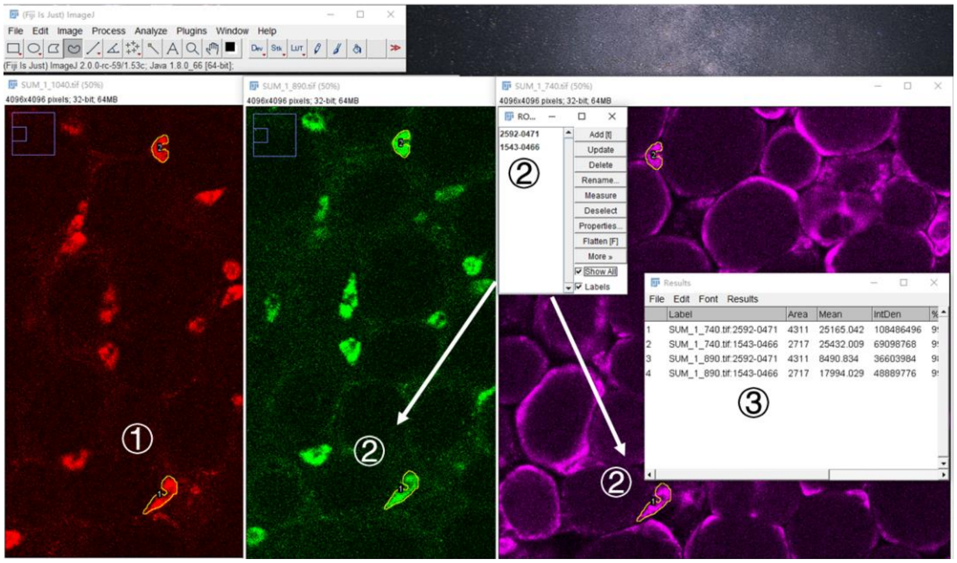

1) Locate macrophages based on lipofuscin-like red autofluorescence, and use the freehand selections tool in Image J to circle the cells with red fluorescence.
2) Apply these ROIs to NAD(P)H and FAD fluorescence images, respectively, and measure the NAD(P)H and FAD intensities of each ROI.
3) Calculate the redox ratio of each cell based on the formula of I_{FAD} / [$I_{NAD(P)H}$ + I_{FAD}], and analyze at least 30 cells' redox ratio.

Calculation of adipocyte redox ratio

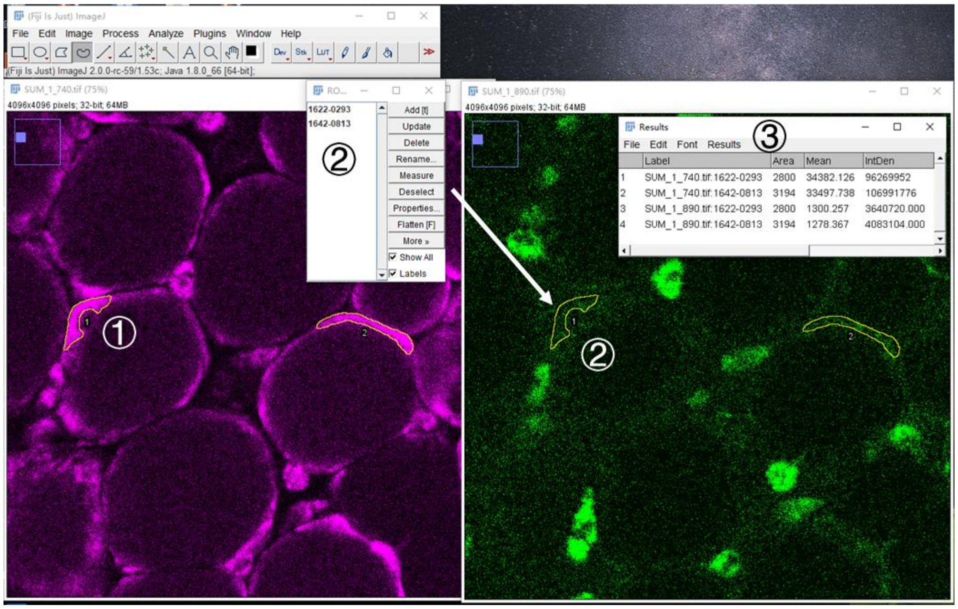

Adipocyte cytoplasm can be visualized in both fluorescence channels, whereas lipids have little detectable signal in the FAD channel [32]. So, the lipids area and macrophage region was excluded when calculating the redox ratio. The steps to analyze the redox ratio of adipocytes are as follows:

1) Circle the adipocyte cytoplasm on the NAD(P)H fluorescence image using the freehand selections tool in Image J.
2) Apply these ROIs to the FAD fluorescence image and measure the NAD(P)H and FAD intensities of each ROI.
3) Calculate the redox ratio of each cell based on the formula of $I_{FAD} / [I_{NAD(P)H} + I_{FAD}]$, and analyze at least 30 cells' redox ratio.

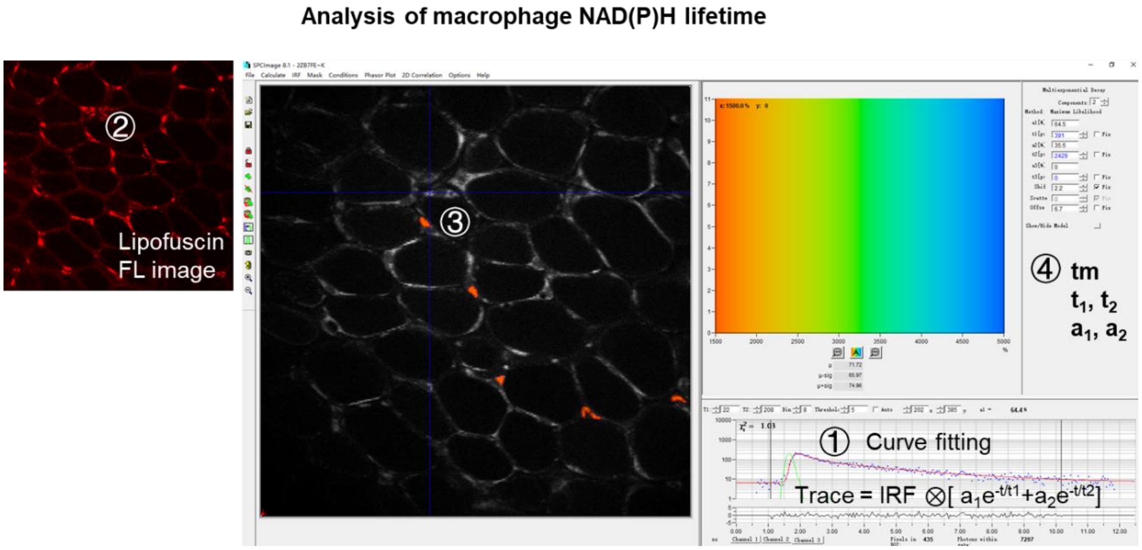

1）Use an IRF convoluted two-component model $f(t) = IRF \otimes (a_1e^{-t/t1} + a_2e^{-t/t2})$ to fit the decay traces.

2）Locate macrophages based on lipofuscin fluorescence image.

3）Circle macrophages on the NAD(P)H fluorescence lifetime image using the "Define mask" tool in SPCimage software.

4) Export the optical metrics, including a_1, t_1, a_2, t_2, and t_m, and analyze at least 30 cells.

Analysis of adipocyte NAD(P)H lifetime

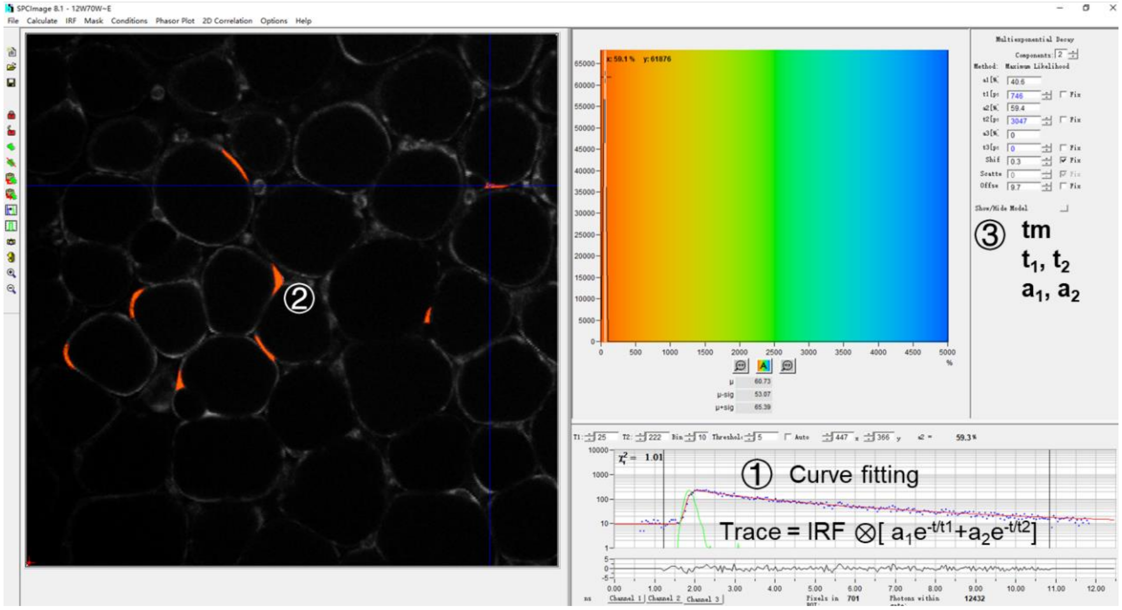

1) Use an IRF convoluted two-component model $f(t) = IRF \otimes (a_1e^{-t/t1} + a_2e^{-t/t2})$ to fit the decay traces.
2) Circle the adipocyte cytoplasm on the NAD(P)H fluorescence lifetime image using the "Define mask" tool in SPCimage software.
3) Export the optical metrics, including a_1, t_1, a_2, t_2, and t_m, and collect at least 30 cells' NAD(P)H lifetime.

Procedures for measuring adipocyte size

Measurement of adipocyte size

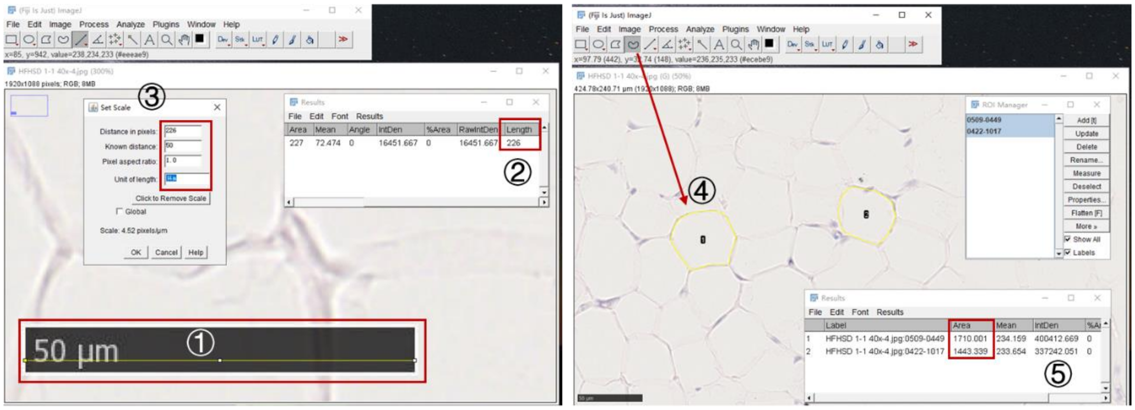

1-3) Make sure that the pixel resolution of the image agrees with the scale bar.
4) Manually circle the adipocyte using the freehand selections tool in Image J.
5) Measure the ROI and obtain the area of adipocytes.

Forest plot of Odds Ratios: Screening the association between optical matrics and diabetes

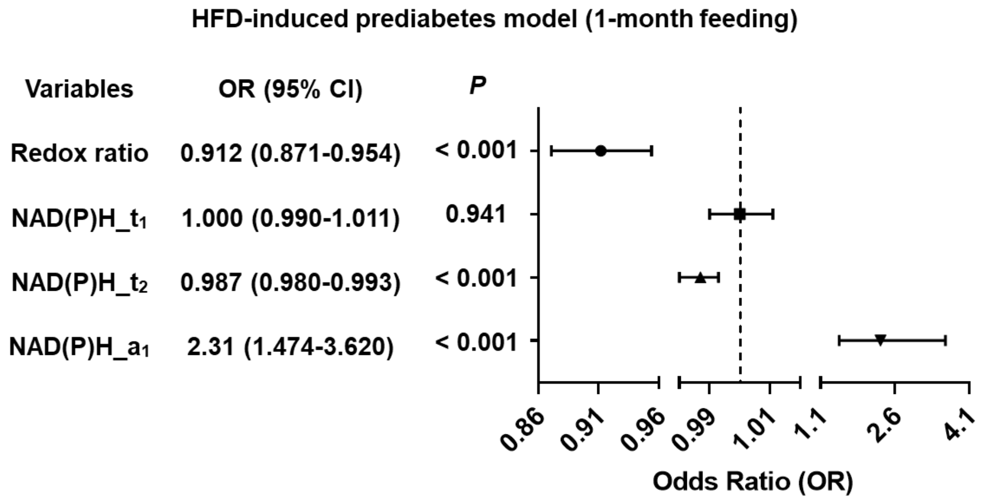

Forest plot showing the association between adipocytes' optical metrics and prediabetes (HFD-induced model, 1 month of feeding).

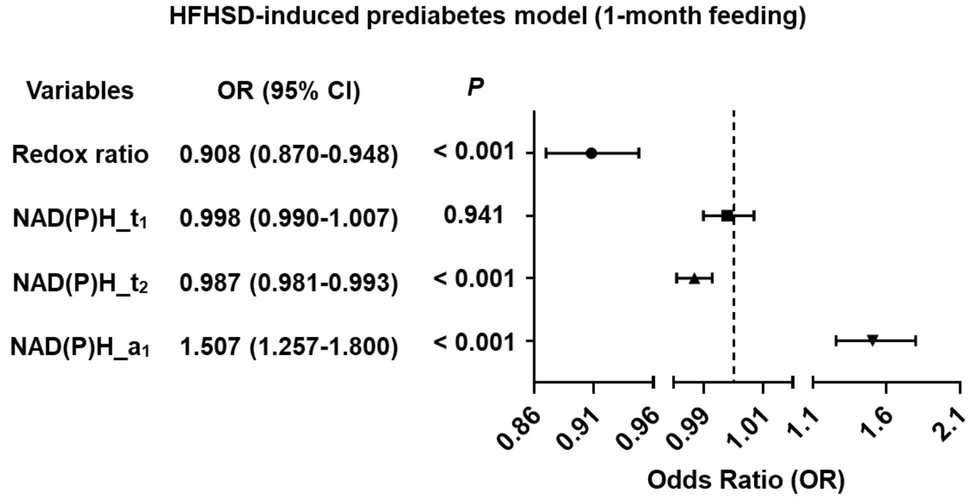

Forest plot showing the association between adipocytes' optical metrics and prediabetes (HFHSD-induced model, 1 month of feeding).

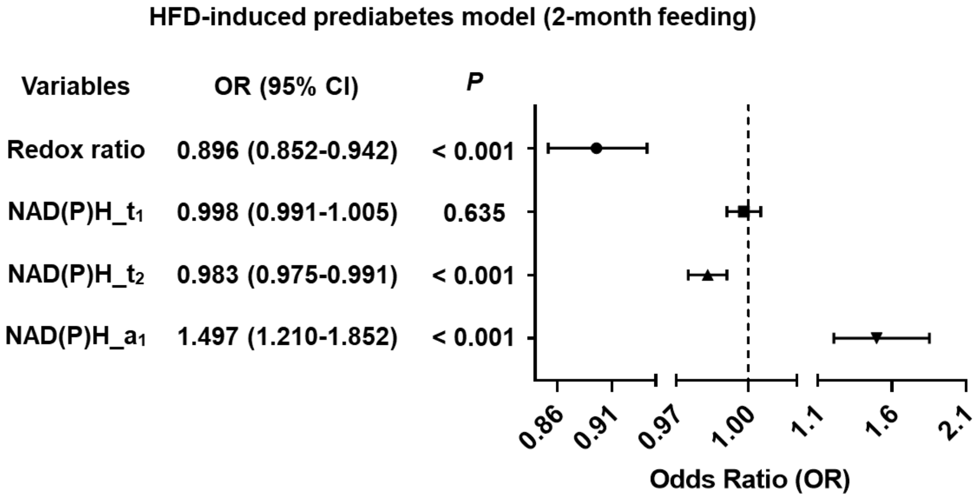

Forest plot showing the association between adipocytes' optical metrics and prediabetes (HFD-induced model, 2 months of feeding).

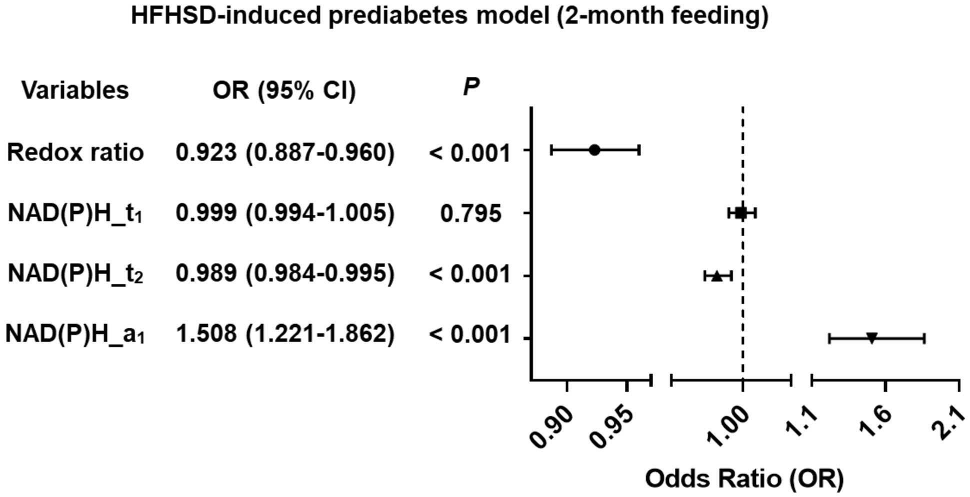

Forest plot showing the association between adipocytes' optical metrics and prediabetes (HFHSD-induced model, 2 months of feeding).

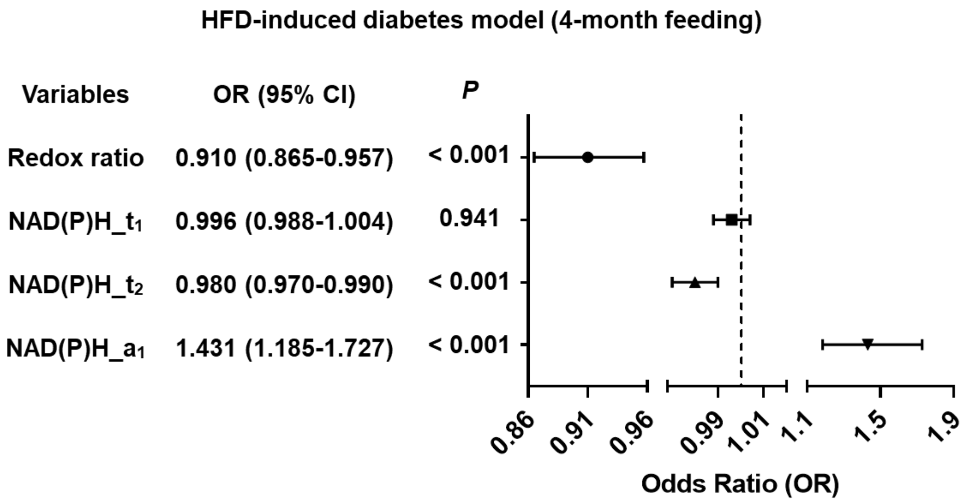

Forest plot showing the association between adipocytes' optical metrics and diabetes (HFD-induced model, 4 months of feeding).

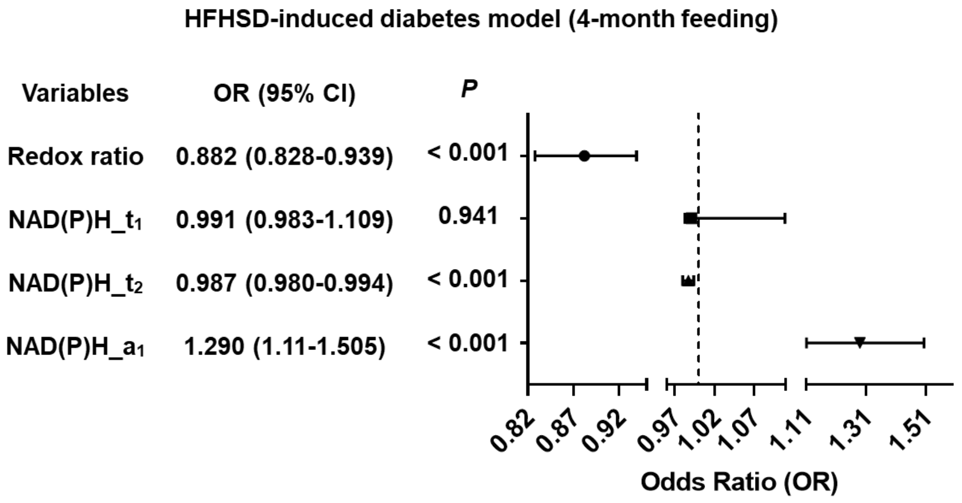

Forest plot showing the association between adipocytes' optical metrics and diabetes (HFHSD-induced model, 4 months of feeding).

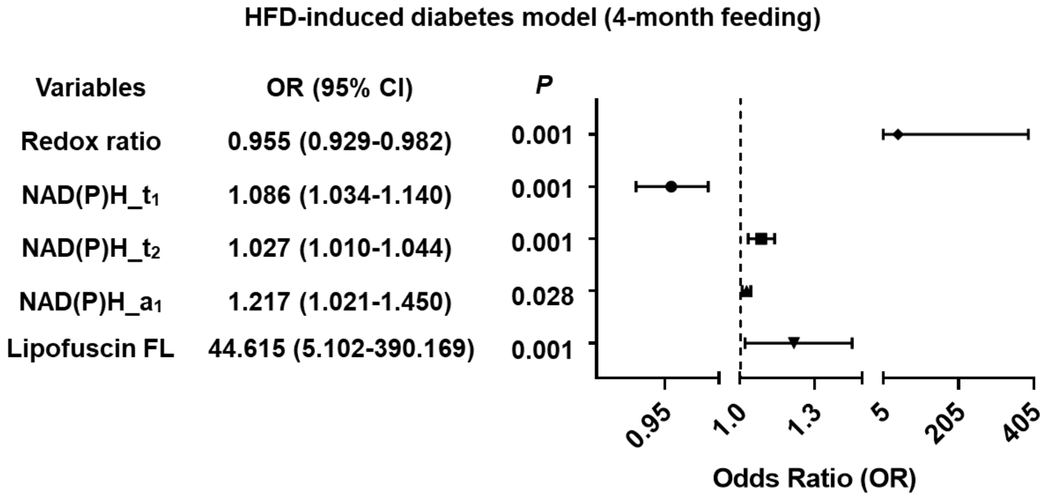

Forest plot showing the association between macrophages' optical metrics and diabetes (HFD-induced model, 4 months of feeding).

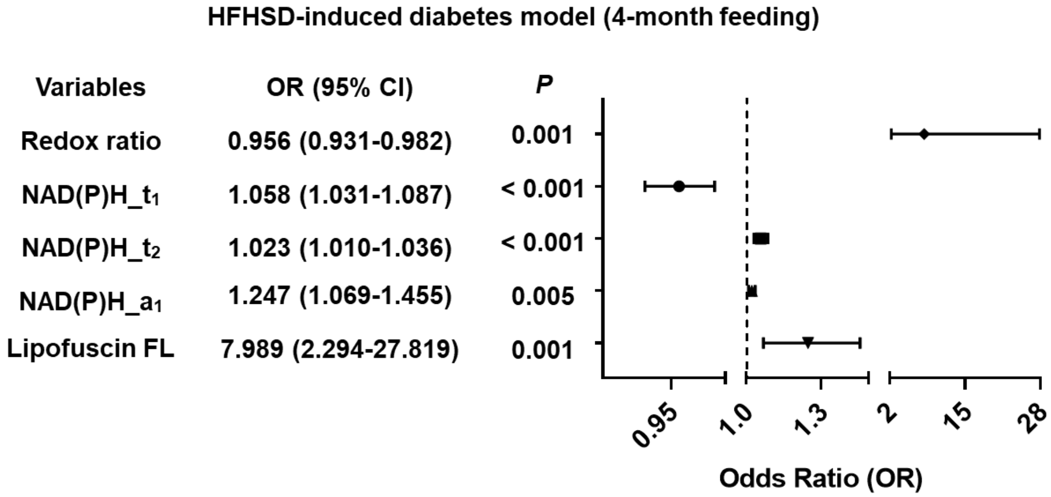

Forest plot showing the association between macrophages' optical metrics and diabetes (HFHSD-induced model, 4 months of feeding).

References

1. Coats BR, Schoenfelt KQ, Barbosa-Lorenzi VC, Peris E, Cui C, Hoffman A, et al. Metabolically activated adipose tissue macrophages perform detrimental and beneficial functions during diet-induced obesity. Cell Rep. 2017; 20: 3149-61.

2. Zhang P, Li T, Wu X, Nice EC, Huang C, Zhang Y. Oxidative stress and diabetes: antioxidative strategies. Front Med. 2020; 14: 583-600.

3. Khan MSH, Hegde V. Obesity and diabetes mediated chronic inflammation: a potential biomarker in alzheimer's disease. J Pers Med. 2020; 10.

4. Bournat JC, Brown CW. Mitochondrial dysfunction in obesity. Curr Opin Endocrinol Diabetes Obes. 2010; 17: 446-52.

5. Catrysse L, van Loo G. Adipose tissue macrophages and their polarization in health and obesity. Cell Immunol. 2018; 330: 114-9.

6. Barbier-Torres L, Fortner KA, Iruzubieta P, Delgado TC, Giddings E, Chen Y, et al. Silencing hepatic MCJ attenuates non-alcoholic fatty liver disease (NAFLD) by increasing mitochondrial fatty acid oxidation. Nat Commun. 2020; 11: 3360.

7. Bapat SP, Whitty C, Mowery CT, Liang Y, Yoo A, Jiang Z, et al. Obesity alters pathology and treatment response in inflammatory disease. Nature. 2022; 604: 337-42.

8. Huang J, Jia Y, Fu T, Viswakarma N, Bai L, Rao MS, et al. Sustained activation of PPARα by endogenous ligands increases hepatic fatty acid oxidation and prevents obesity in ob/ob mice. FASEB J. 2012; 26: 628-38.

9. Hua Q, Han Y, Zhao H, Zhang H, Yan B, Pei S, et al. Punicalagin alleviates renal injury via the gut-kidney axis in high-fat diet-induced diabetic mice. Food Funct. 2022; 13: 867-79.

10. Bao J, Liang Z, Gong X, Yu J, Xiao Y, Liu W, et al. High fat diet mediates amyloid-β cleaving enzyme 1 phosphorylation and SUMOylation, enhancing cognitive impairment in APP/PS1 Mice. J Alzheimers Dis. 2022; 85: 863-76.

11. Brahma MK, Ha CM, Pepin ME, Mia S, Sun Z, Chatham JC, et al. Increased glucose availability attenuates myocardial ketone body utilization. J Am Heart Assoc. 2020; 9: e013039.

12. Mayer AE, Löffler MC, Loza Valdés AE, Schmitz W, El-Merahbi R, Viera JT, et al. The kinase PKD3 provides negative feedback on cholesterol and triglyceride synthesis by suppressing insulin signaling. Sci Signal. 2019; 12.

13. Fraulob JC, Ogg-Diamantino R, Fernandes-Santos C, Aguila MB, Mandarim-de-Lacerda CA. A mouse model of metabolic syndrome: insulin resistance, fatty liver and non-alcoholic fatty pancreas disease (NAFPD) in C57BL/6 mice fed a high fat diet. J Clin Biochem Nutr. 2010; 46: 212-23.

14. Avtanski D, Pavlov VA, Tracey KJ, Poretsky L. Characterization of inflammation and insulin resistance in high-fat diet-induced male C57BL/6J mouse model of obesity. Animal Model Exp Med. 2019; 2: 252-8.

15. Selvin E, Rawlings AM, Bergenstal RM, Coresh J, Brancati FL. No racial differences in the association of glycated hemoglobin with kidney disease and cardiovascular outcomes. Diabetes Care. 2013; 36: 2995-3001.

16. Selvin E. Are there clinical implications of racial differences in HbA1c? A difference, to be a difference, must make a difference. Diabetes Care. 2016; 39: 1462-7.

17. Surwit RS, Kuhn CM, Cochrane C, McCubbin JA, Feinglos MN. Diet-induced type 2 diabetes in C57BL/6J mice. Diabetes. 1988; 37: 1163-7.

18. Tabák AG, Jokela M, Akbaraly TN, Brunner EJ, Kivimäki M, Witte DR. Trajectories of glycaemia, insulin sensitivity, and insulin secretion before diagnosis of type 2 diabetes: an analysis from the Whitehall II study. Lancet. 2009; 373: 2215-21.

19. Hulman A, Simmons RK, Brunner EJ, Witte DR, Færch K, Vistisen D, et al. Trajectories of glycaemia, insulin sensitivity and insulin secretion in South Asian and white individuals before diagnosis of type 2 diabetes: a longitudinal analysis from the Whitehall II cohort study. Diabetologia. 2017; 60: 1252-60.

20. Tabák AG, Herder C, Rathmann W, Brunner EJ, Kivimäki M. Prediabetes: a high-risk state for diabetes development. Lancet. 2012; 379: 2279-90.

21. Kim M, Song K, Kim YS. Alantolactone improves prolonged exposure of interleukin-6-induced skeletal muscle inflammation associated glucose intolerance and insulin resistance. Front Pharmacol. 2017; 8: 405.

22. Zou C, Wang Y, Shen Z. 2-NBDG as a fluorescent indicator for direct glucose uptake measurement. J Biochem Biophys Methods. 2005; 64: 207-15.

23. Asakawa H, Tokunaga K, Kawakami F. Relationship of leptin level with metabolic disorders and hypertension in Japanese type 2 diabetes mellitus patients. J Diabetes Complications. 2001; 15: 57-62.

24. Uslu S, Kebapçi N, Kara M, Bal C. Relationship between adipocytokines and cardiovascular risk factors in patients with type 2 diabetes mellitus. Exp Ther Med. 2012; 4: 113-20.

25. Abdella NA, Mojiminiyi OA, Moussa MA, Zaki M, Al Mohammedi H, Al Ozairi ES, et al. Plasma leptin concentration in patients with Type 2 diabetes: relationship to cardiovascular disease risk factors and insulin resistance. Diabet Med. 2005; 22: 278-85.

26. López-Jaramillo P, Gómez-Arbeláez D, López-López J, López-López C, Martínez-Ortega J, Gómez-Rodríguez A, et al. The role of leptin/adiponectin ratio in metabolic syndrome and diabetes. Horm Mol Biol Clin Investig. 2014; 18: 37-45.

27. Petersen KF, Befroy D, Dufour S, Dziura J, Ariyan C, Rothman DL, et al. Mitochondrial dysfunction in the elderly: possible role in insulin resistance. Science. 2003; 300: 1140-2.

28. Kelley DE, He J, Menshikova EV, Ritov VB. Dysfunction of mitochondria in human skeletal muscle in type 2 diabetes. Diabetes. 2002; 51: 2944-50.

29. Coughlan KA, Valentine RJ, Ruderman NB, Saha AK. AMPK activation: a therapeutic target for type 2 diabetes? Diabetes Metab Syndr Obes. 2014; 7: 241-53.

30. Hu Y, Chen Y, Ding L, He X, Takahashi Y, Gao Y, et al. Pathogenic role of diabetes-induced PPAR-α down-regulation in microvascular dysfunction. Proc Natl Acad Sci U S A. 2013; 110: 15401-6.

31. Mootha VK, Lindgren CM, Eriksson KF, Subramanian A, Sihag S, Lehar J, et al. PGC-1α-responsive genes involved in oxidative phosphorylation are coordinately downregulated in human diabetes. Nat Genet. 2003; 34: 267-73.

32. Alonzo CA, Karaliota S, Pouli D, Liu Z, Karalis KP, Georgakoudi I. Two-photon excited fluorescence of intrinsic fluorophores enables label-free assessment of adipose tissue function. Sci Rep. 2016; 6: 31012.
